# Toward Bright Mid-Infrared Emitters: Thick-Shell n-Type HgSe/CdS Nanocrystals

**DOI:** 10.1021/jacs.1c09858

**Published:** 2021-11-09

**Authors:** Ananth Kamath, Christopher Melnychuk, Philippe Guyot-Sionnest

**Affiliations:** Department of Chemistry and the James Franck Institute, The University of Chicago, 929 East 57th Street, Chicago, Illinois 60637, United States

## Abstract

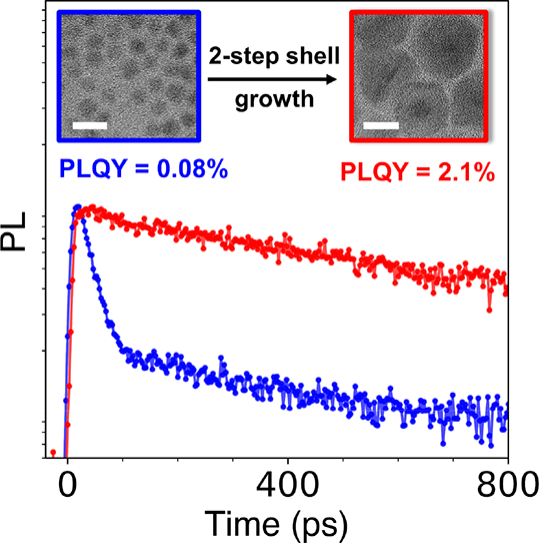

A procedure is developed for the growth of thick, conformal CdS shells that preserve the optical properties of 5 nm HgSe cores. The n-doping of the HgSe/CdS core/shell particles is quantitatively tuned through a simple postsynthetic Cd treatment, while the doping is monitored via the intraband optical absorption at 5 μm wavelength. Photoluminescence lifetime and quantum yield measurements show that the CdS shell greatly increases the intraband emission intensity. This indicates that decoupling the excitation from the environment reduces the nonradiative recombination. We find that weakly n-type HgSe/CdS are the brightest solution-phase mid-infrared chromophores reported to date at room temperature, achieving intraband photoluminescence quantum yields of 2%. Such photoluminescence corresponds to intraband lifetimes in excess of 10 ns, raising important questions about the fundamental limits to achievable slow intraband relaxation in quantum dots.

## Introduction

The control of intraband carrier relaxation in semiconductor quantum dots (QDs) is a longstanding topic of interest due to its central role in QD optoelectronic technologies.^1−3^ In applications utilizing the interband emission of light, such as conventional QD lasers and LEDs, fast intraband relaxation is desired. In contrast, other applications such as solar energy harvesting utilizing hot-carrier extraction and carrier multiplication are significantly aided when intraband relaxation is slow.^4−6^ Slow intraband relaxation is also required when intraband transitions, typically between the two lowest quantized conduction levels 1S_e_ and 1P_e_, are utilized directly for light emission and detection such as in infrared optoelectronics.^7−12^ It is therefore of broad practical importance to understand and control intraband relaxation rates.

Because of the large energy spacing between QD conduction states, it was initially believed that electronic relaxation should be very slow due to the low expected rate of multiphonon emission across such gaps. This is known as the “phonon bottleneck” effect.^13^ Electronic phonon bottlenecks are rarely observed in practice, however, because electrons can undergo subpicosecond relaxation by coupling to a valence hole in a process known as Auger cooling.^4,14−17^ Experiments that inhibit Auger cooling, either by hole localization^18−20^ or by n-doping,^9,21^ accomplish slower relaxation rates which are often attributed to Forster-like near-field energy transfer involving surface ligand vibrations.^9,18,19,22,23^ This usually produces intraband lifetimes of tens to hundreds of picoseconds.^9,19−21^ Prior to the present work, the longest reported intraband lifetime at 5 μm (2000 cm^–1^) was 1.5 ns in a thick-shell CdSe/ZnS/ZnSe/CdSe QD heterostructure passivated by hole-extracting ligands.^18^

Recent atomistic simulations^24^ using a semiclassical electron–phonon coupling framework^25^ predict that an ∼1 ns lifetime is the fundamental upper limit due to phonon processes intrinsic to all nanocrystals. Fully quantum-mechanical models, however, imply that much longer microsecond lifetimes are attainable when electron–phonon coupling and lattice anharmonicity are small, as in II–VI semiconductors.^26,27^ Furthermore, molecular dynamics simulations and neutron scattering experiments have suggested the presence of strong surface-derived anharmonicities which could fundamentally limit intraband lifetimes to subnanosecond levels.^28,29^ There is consequently a substantial uncertainty regarding the basic limits on maximum achievable intraband lifetimes. A natural experimental test would be to examine the intraband lifetime in a strongly confined QD where Auger cooling, nonradiative energy transfer, and other nonphonon relaxation mechanisms are minimized.

As a first step to address this issue, we report here the synthesis and spectroscopy of thick-shell n-type HgSe/CdS core/shell QDs. HgSe QDs are a convenient system for studies of intraband electronic relaxation due to their air-stable n-doping,^7,9,30^ intraband photoluminescence, and absence of Auger electron cooling.^9^ They are also investigated for mid-infrared optoelectronics due to suppressed multicarrier Auger recombination,^9^ solution processability, and greatly reduced material costs relative to epitaxial materials.^7,31^ Prior studies focused on HgSe QDs with no shell or thin shells,^9,32−34^ and the intraband photoluminescence quantum yields (PLQYs) remained around 0.1%,^34^ indicating subnanosecond intraband nonradiative lifetimes.^9^ When growing core/shell nanocrystals, high temperatures are usually needed to promote a compact shell growth. Established procedures for growing thick CdE (E = S/Se) shells on CdSe,^35−39^ PbS,^40^ PbSe,^41,42^ ZnSe,^43^ and InP^44^ QDs demand temperatures in the 240–300 °C range. This substantially exceeds the alloying temperature for HgSe/CdS^34^ and motivates the development of a new synthetic protocol. Although HgSe/CdS can be grown by colloidal atomic layer deposition (cALD), such procedures are prone to substantial homogeneous nucleation of CdS nanocrystals after a few shell layers.^33,34^

In this work, the synthesis of thick-shell HgSe/CdS QDs under milder conditions is accomplished via a two-step growth procedure utilizing highly reactive single-source precursors.^45−47^ We demonstrate increasing intraband lifetimes and PLQYs with increasing shell thickness, resulting in the highest photoluminescence efficiencies and longest intraband lifetimes reported to date at 2000 cm^–1^ (5 μm).

## Results and Discussion

### Synthesis of Thick-Shell HgSe/CdS Core/Shell QDs

All syntheses were performed on 4.8 ± 0.5 nm diameter HgSe QD cores to obtain intraband photoluminescence peaked near 2000 cm^–1^, the spectral region of interest for mid-infrared photodetectors and light sources. The HgSe/CdS synthesis begins with an initial CdS growth at 80 °C utilizing cadmium bis(phenyldithiocarbamate) (Cd(PDTC)_2_), a highly reactive single-source precursor previously used for the low-temperature growth of CdSe/CdS nanobelts.^48^ Through a kinetics study, we found that Cd(PDTC)_2_ decomposes to CdS at temperatures above 60 °C and that the optimal temperature for shell growth is 80 °C (Supporting Information section 1J). This single-step synthesis avoids complications associated with multistep room-temperature cALD,^33,34^ and it has the added advantage of easy scalability. After growth of a 0.6 nm thick CdS layer, the QDs exhibited thermal stability (Figure S1F) such that thicker CdS shells could be subsequently grown by using cadmium bis(diethyldithiocarbamate) (Cd(DEDTC)_2_) at 220 °C. This temperature was found to provide a good balance between minimizing interfacial core/shell alloying and promoting quasi-spherical shell growth (Supporting Information section 1I).^34^

**Figure 1:**
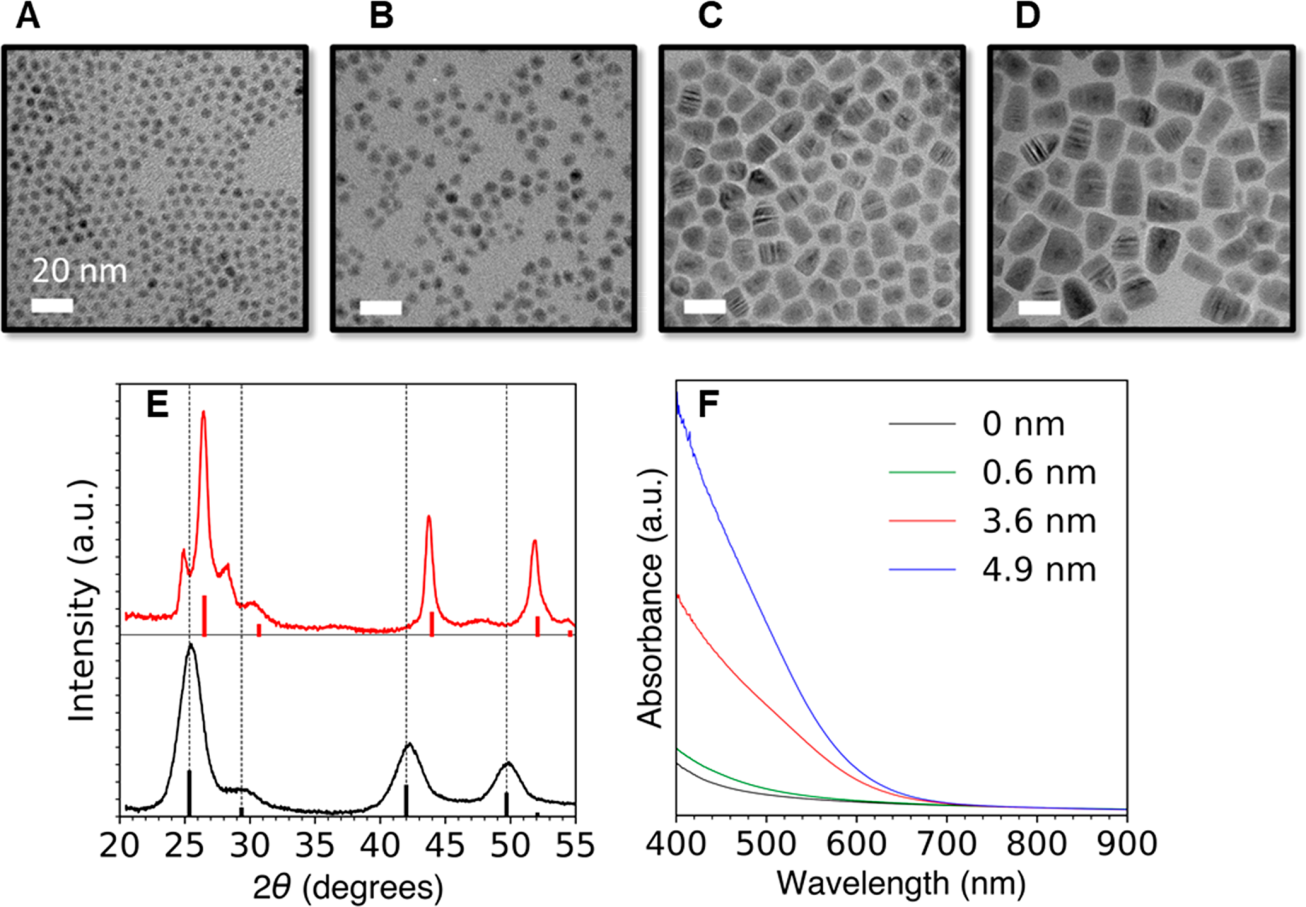
(A–D) TEM images of HgSe/CdS QDs with a core diameter of (A) 4.8 ± 0.6 nm and core/shell diameter of (B) 6.0 ± 1.2 nm, (C) 11.5 ± 1.4 nm, and (D) 15.3 ± 2.7 nm. (E) powder XRD spectra of (black) 4.8 nm HgSe and (red) 19 nm HgSe/CdS QDs. Black and red solid bars indicate the XRD peaks of bulk zincblende HgSe and CdS, respectively. (F) Absorption spectra of HgSe and HgSe/CdS QDs with the indicated shell thicknesses, normalized to HgSe core absorption at 808 nm.

HgSe QDs display the cubic zincblende structure,^7^ while CdS may grow along either cubic zincblende or hexagonal wurtzite structures depending on the synthetic conditions.^45^ The growth of a wurtzite shell on a zincblende core produces polypods^42,49−51^ which can promote fast nonradiative relaxation.^42^ It is therefore necessary to grow the CdS shell along a zincblende structure, and we accomplish this through an appropriate choice of ligands. Growth of CdS has been previously reported to occur along a zincblende structure when cadmium carboxylates are used as ligands.^52^ Indeed, during syntheses with only Cd(DEDTC)_2_ and amine ligands, we observe a significant wurtzite shell component, and the CdS shell begins to develop tetrapodal arms (Figure S1I(C,D)).^47^ We find that cadmium oleate works as a good ligand to promote CdS growth along a zincblende structure (Figure S1I(I,K)), allowing growth of a thick and uniform shell (Figure 1 and Figure S1I(E,F)). We note that the cadmium oleate should contain no residual oleic acid, as even slight amounts lead to QDs with poor intraband photoluminescence.

Transmission electron microscopy (TEM) images indicate that the nanocrystal diameter grows from 4.8 to 15.3 nm during 30 min of CdS growth using Cd(DEDTC)_2_ (Figure 1A–D). At diameters above 12 nm, they begin growing as bullet shapes. The average diameter was measured from the TEM images as a geometric mean of the short diameters and long axis (Supporting Information section 3C). The pXRD in Figure 1E indicates that thick CdS shell growth occurs predominantly in a zincblende structure. Wurtzite peaks along the (100), (101), and (103) planes (at 25°, 29°, and 48°, respectively) are observed, which accounts for 25% of the total signal (Figure S2D). The absorption spectra (Figure 1F) show the onset of a strong visible absorption due to the CdS shell, while the HgSe interband and intraband absorptions at 6000 and 2500 cm^–1^ are retained (Figure S1G). This demonstrates that the integrity of the cores is maintained after the shell growth.

Under these synthetic conditions, we observe that the nanocrystal sizes measured by TEM are larger than predicted by the precursor amount added. We also observe a red tail in the visible absorption beyond the CdS band edge, whose intensity varies with synthetic conditions. We believe that during the initial stages of the thick-shell HgSe/CdS synthesis a fraction of the thin shell HgSe/CdS QDs dissolve and deposit upon the remaining QDs as a HgCdSSe shell. This would explain the red tail and larger core/shell sizes than calculated from the amounts of precursors added. A batch-to-batch variability is observed in the extent of dissolution, and it also depends on the heating rate. The benefit, however, is the promotion of compact thick shell growth. We attribute this to reduced strain at the core/shell interface, with a possible gradient alloying. We observe no noticeable effect on the intraband and interband absorptions (see section 3A in the Supporting Information for more details).

### Measurement and Chemical Control of 1S_e_ Occupancy

**Figure 2:**
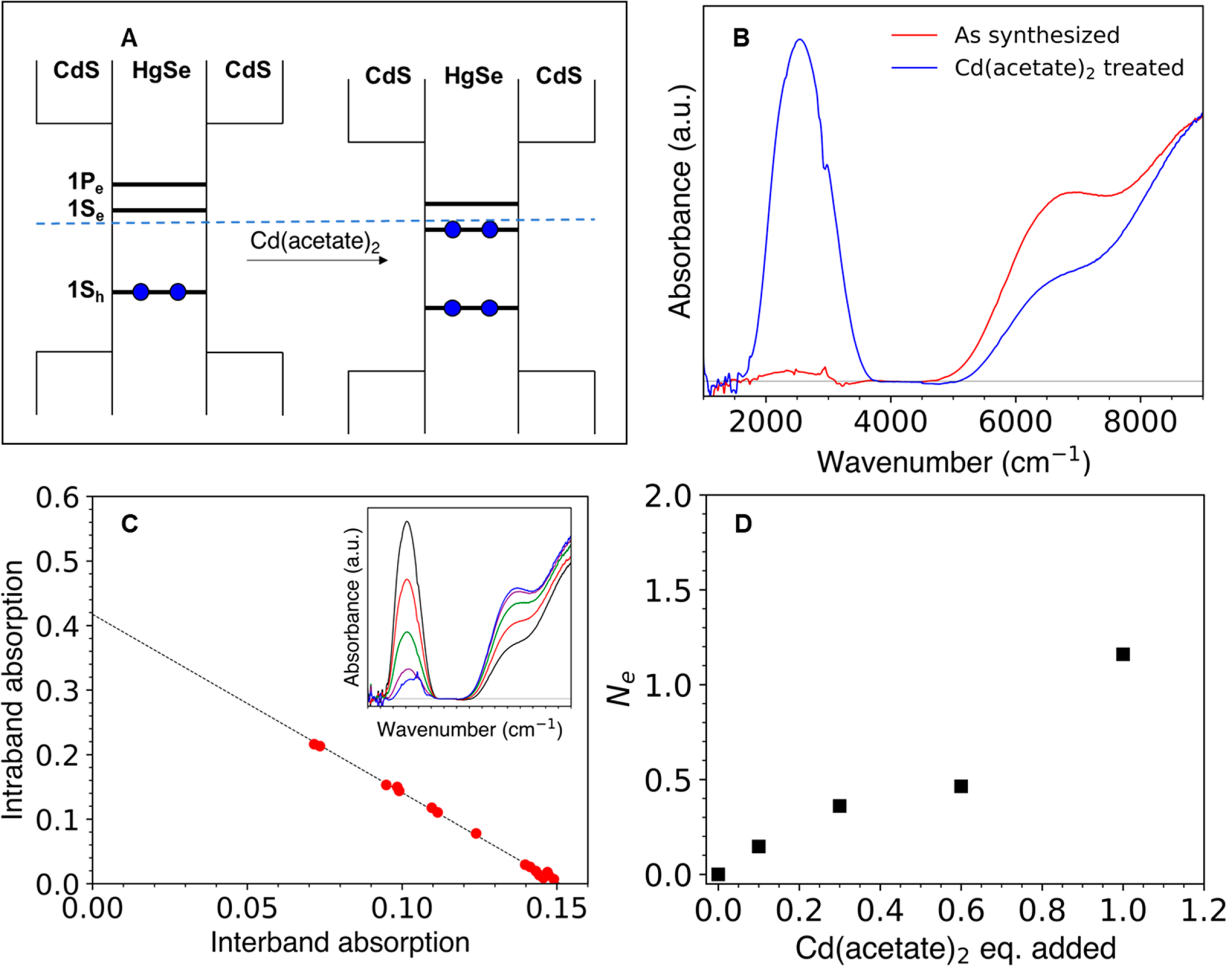
Control of the 1Se occupancy (*N*_e_) of HgSe/CdS QDs. (A) Cartoon of surface dipole n-doping mechanism in HgSe/CdS QDs. The ambient Fermi level is indicated by the dashed blue line. (B) FTIR spectra of HgSe/CdS QDs (red) after synthesis and (blue) after treatment with cadmium acetate. Absorptions from the ligands are subtracted for clarity. (C) Plot of intraband vs interband absorption of HgSe/CdS QDs with different doping levels. Inset shows the FTIR spectra (after subtraction of ligand absorption) of HgSe/CdS QDs after titrating with I_2_. (D) Average 1S_e_ occupancy (*N*_e_) as a function of the surface equivalents of cadmium acetate added (see Supporting Information section 1H).

Although HgSe QDs are n-type under ambient conditions with electrons in 1S_e_,^9,30^ CdS shell growth tends to remove the natural doping. One must therefore n-dope the HgSe/CdS QDs after synthesis to turn on the 1S_e_–1P_e_ intraband transition. Several methods are commonly employed to dope QDs including incorporation of aliovalent impurities,^53−55^ surface oxidation,^56,57^ changing surface dipole through ligand exchange,^7,58,59^ charge injection through electrochemistry,^60−65^ or redox agents.^66−68^ Here we employ a surface dipole modification which, as depicted in Figure 2A, shifts the absolute positions of the QD energy levels relative to a fixed environmental Fermi level. We denote the QDs with 0, 1, and 2 electrons in the 1S_e_ state as 1S_e_(0), 1S_e_(1), and 1S_e_(2), respectively. Because the 1S_e_ occupancy (doping) affects absorption and photoluminescence, it is necessary to control and characterize the doping.

The doping of HgSe/CdS QDs after synthesis is sensitive to the quantities of cadmium oleate ligand and CdS precursor utilized during the shell growth. Cadmium oleate promotes n-doping, attributed to the introduction of a positive surface species and inward-pointing surface dipole (Figure 2A,B). The Cd(DEDTC)_2_ precursor evolves H_2_S during the reaction^46,69^ and likely deposits S^2–^ on the QD surface, which reduces the n-doping. To control the doping, we developed a procedure in which we first oxidize the HgSe/CdS QDs by treatment with ammonium sulfide at 40 °C to achieve a 1S_e_ occupancy (*N*_e_) of ∼0 (Supporting Information section 1H). The QDs are subsequently treated with cadmium acetate at 180 °C, and the doping level can be tuned by changing the cadmium acetate amount (Figure 2D).

At low dopings, the intraband absorption is shadowed by the surface ligand absorption (Figure S2C-1(D)). To obtain quantitative optical measurement of the doping, we used a solution of molecular iodine in tetrachloroethylene (TCE) as a temporary oxidizing agent that does not change the ligand absorption.^70^ A subtractive procedure then eliminated the ligand absorption, giving a clean intraband absorption required for optical doping determination (Figure S2C-1). The interband and intraband absorptions are linearly related as would be expected from a transfer of oscillator strength, as shown in Figure 2C (see Figure S3E for a comparison of oscillator strengths). From a linear fit to the intraband–interband trend, we determine the *N*_e_ of any QD sample from the ratio of the intraband peak absorbance to the *y*-intercept.

### Photoluminescence and Nonradiative Relaxation

Photoluminescence spectra of the QDs were recorded in TCE solution by excitation with an 808 nm laser, and the measurement details may be found in the Supporting Information. Photoexcitation of 1S_e_(0) QDs can only lead to interband emission. On the other hand, photoexcitation of the 1S_e_(1) and 1S_e_(2) HgSe/CdS QDs can lead to formation of a hole in either the valence band or the conduction band. Because of fast hole cooling, likely by hole Auger cooling^4^ in n-doped HgSe QDs, the hole relaxes to the 1S_e_ state within a few picoseconds.^9^ This leads to only intraband emission in n-doped 1S_e_(1) and 1S_e_(2) QDs, irrespective of whether the photoexcitation is from the valence or conduction band (Kasha’s rule).^71^

**Figure 3:**
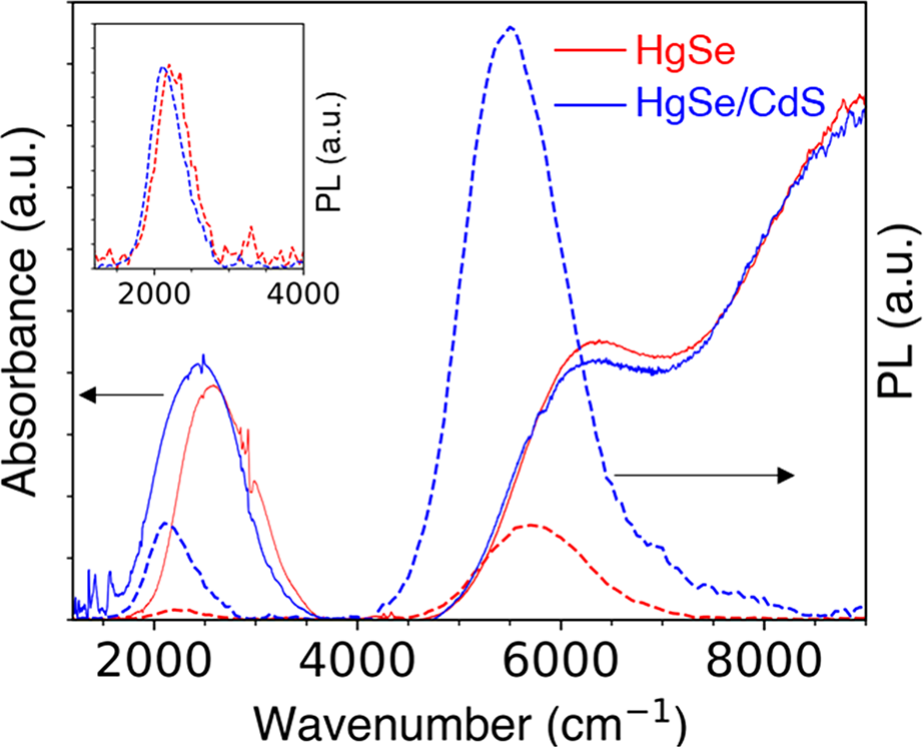
Absorption (solid lines) and PL (dashed lines) spectra of (red) 4.8 ± 0.6 nm HgSe and (blue) 10.2 ± 1.6 nm HgSe/CdS with *N*_e_ = 0.5. The ligand absorptions have been subtracted for clarity. The inset shows a comparison of intraband PL spectra with the HgSe PL scaled 10×.

As shown in Figure 3, the absorption and photoluminescence (PL) spectral peaks of HgSe/CdS QDs show a negligible red-shift compared to HgSe QDs, supporting the strong type I core/shell band alignment. The PL quantum efficiencies (PLQE), defined here as the global fraction of interband or intraband photons emitted per photon absorbed, do not directly inform on the nonradiative relaxation because they depend on the doping level. We therefore determine the contributions from 1S_e_(0), 1S_e_(1), and 1S_e_(2) populations to the PLQE and normalize by the relative populations to determine the absolute PL quantum yields (PLQYs) of the three species. The interband emission is expected to primarily arise from 1S_e_(0) QDs as noted earlier, while to a first approximation the intraband emission should be proportional to the sum of 1S_e_(1) and 1S_e_(2) populations.

**Figure 4:**
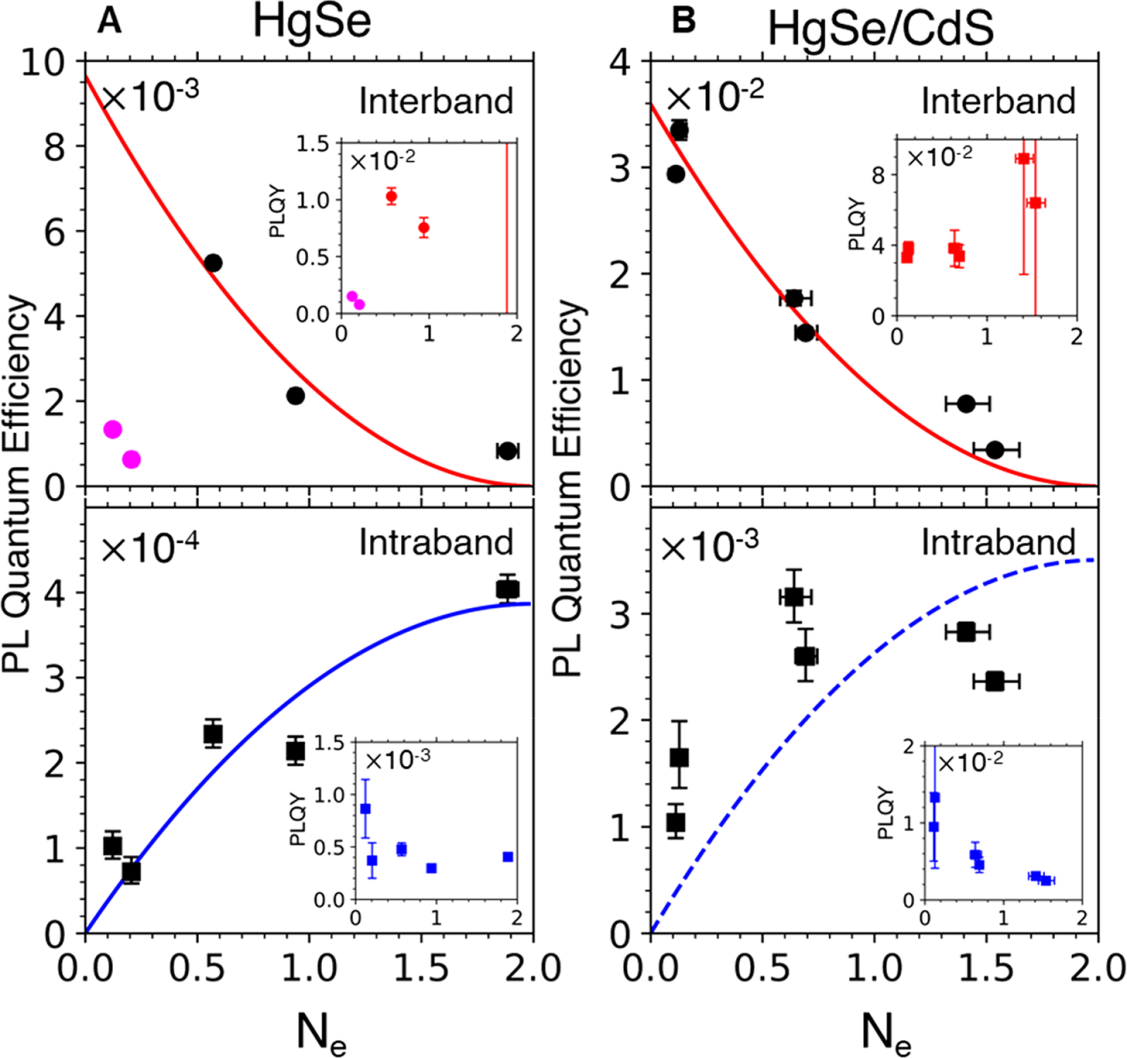
PLQE vs *N*_e_ for (A) 4.8 ± 0.6 nm HgSe QDs and (B) 11.6 ± 1.6 nm HgSe/CdS QDs. The red curves are fits from the 1S_e_(0) binomial population. The two samples (pink points) with the lowest dopings for HgSe in (A) have a low interband PL due to the poor surface passivation and are excluded from the fit. The blue curve in (A) is fit assuming same PLQYs for 1S_e_(1) and 1S_e_(2) populations. The data in (B) fit poorly to a constant PLQY (dashed blue). The insets show the PLQY calculated by normalizing the PLQE to 1S_e_(0) population (for interband) and the sum of 1S_e_(1) and 1S_e_(2) populations (for intraband).

Intraband and interband PLQE data at different ensemble *N*_e_ are shown for 4.8 nm diameter HgSe (Figure 4A) and for similar HgSe with 3.4 nm CdS shell thickness (Figure 4B). *N*_e_ at a fixed Fermi level is determined by Fermi–Dirac statistics, while the fraction of QDs with 0, 1, or 2 electrons in the 1S_e_ state is expected to follow a binomial distribution if there is no significant electronic correlation (Supporting Information section 3D). The interband emissions in Figure 4 are well-fit by the 1S_e_(0) occupancy, which is consistent with Kasha’s rule. The fitting gives interband PLQYs of (9.5 ± 1.0) × 10^–3^ and (3.6 ± 0.1) × 10^–2^ for HgSe and HgSe/CdS, respectively. Likewise, the intraband PLQE for HgSe fits well to a sum of 1S_e_(1) and 1S_e_(2) populations with a PLQY of (3.8 ± 0.5) × 10^–4^ for both species.

In contrast, the intraband PLQE of HgSe/CdS cannot be fit as well by assuming a constant intraband PLQY (Figure 4B, dashed blue). The decreasing intraband PLQY of HgSe/CdS with the doping (Figure 4B, inset) suggests the presence of a nonradiative pathway that changes with the doping. This interpretation is qualitatively supported by PL lifetime measurements on HgSe/CdS QDs which show a faster intraband decay at higher doping levels (Figure S2E). One possible mechanism is the presence of defect states in the CdS shell close to the 1S_e_ of HgSe which would be occupied upon surface dipole-induced energy level shifting (Figure 2A). Indeed, bulk CdS is known to exhibit deep electronic defect states which can be infrared active.^72−74^ The filling of these defect states could introduce a nonradiative pathway by either hole trapping or resonant energy transfer (Figure S3F).

**Figure 5:**
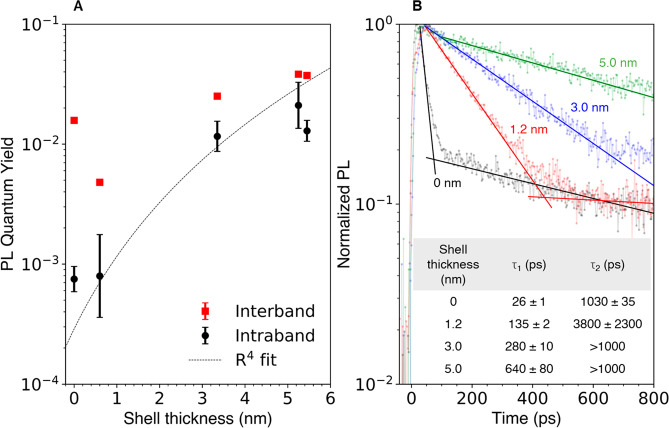
(A) (red squares) 1S_e_(0) interband and (black circles) 1S_e_(1) intraband PLQY of HgSe/CdS QDs with different shell thicknesses. Except the cores (with *N*_e_ ∼ 1), all samples had *N*_e_ < 0.2. The intraband PLQY data are fit to an *R*^4^ function (black dotted line), physically motivated by the expected trend from Forster-type nonradiative relaxation to surface vibrations. (B) Intraband PL lifetime traces for HgSe (*N*_e_ ∼ 2) and HgSe/CdS QDs (*N*_e_ < 0.5). All traces fit well to biexponentials, while the long lifetimes for the thickest samples are too long to determine.

To minimize the doping-dependent nonradiative effects and investigate the underlying relaxation mechanisms, we further examined the influence of CdS shell thickness for QDs with *N*_e_ < 0.2. These data are shown in Figure 5. Growth of a thick shell leads to a weak increase in the interband PLQY, with the exception of the thin shell HgSe/CdS QDs (Figure 5A, 0.6 nm shell thickness). These QDs display a relatively poor interband PLQY of 0.5% due to the low temperature shell synthesis, which increases to >2% on annealing at 220 °C (Figure S1F(B)). The weak increase of interband PLQY with shell thickness is qualitatively similar to prior works which reported saturation of interband HgSe/CdS PLQY at moderate shell thicknesses.^33,34^ In contrast, the intraband PLQY exhibits a 30-fold increase over the same shell thickness range (Figure 5A).

The intraband radiative lifetime τ_R_ of HgSe QDs can be calculated from eq 1(75−78) where *p* is the transition dipole moment, ω is the angular frequency, ε_0_ is the vacuum permittivity, *c* is the vacuum speed of light, ℏ is the reduced Planck constant, and *F* is a dielectric factor:
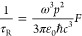
1Due to of the strong type I core/shell band alignment, the emission frequency and transition dipole do not change appreciably upon shell growth (Figure 3). Using material parameters discussed in section 3B of the Supporting Information, the intraband radiative lifetime τ_R_ for HgSe QDs emitting at 2050 cm^–1^ (5 μm) is calculated to be 900 ± 300 ns. Growth of a CdS shell changes the dielectric screening, leading to a radiative lifetime of 700 ± 160 ns for thick shell HgSe/CdS QDs. The PLQY is given by
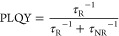
2and the average nonradiative lifetimes τ_NR_ calculated from the measured PLQY are then expected to vary from 700 ps in the HgSe core to 15 ns in HgSe/CdS with 15 nm diameter (Supporting Information section 3B). This increasing trend is clearly supported by transient PL measurements, although they exhibit multiexponential behavior, as shown in Figure 5B. The lifetime data for the HgSe cores are similar to those reported previously,^9^ with two nonradiative relaxation times of 26 ± 1 and 1030 ± 35 ps. About 90% of the time-integrated PL comes from the 1030 ps decay, and the overall transient behavior is consistent with typical PLQYs of the HgSe cores. Shell growth leads to a 25-fold lengthening of the fast lifetime component, while the expected lengthening of the slow lifetime component is not resolved on the time scale of these measurements. Overall, the PL data indicate that thick CdS shells substantially lengthen average intraband nonradiative lifetimes τ_NR_ into the nanosecond regime.

The intraband lifetime derived from the PLQY is at least an order of magnitude longer than the ∼1 ns phonon-mediated lifetime limit predicted by semiclassical simulations.^25^ The near-field energy transfer mechanism^18,19^ predicts that the nonradiative rate should scale with the total nanocrystal radius *R* as *R*^–4^, while phonon-mediated relaxation should be independent of shell thickness and relaxation associated with surface anharmonicity should be strongly reduced even at small type I shell thicknesses. The data of Figure 5A suggest that energy transfer remains the dominant nonradiative mechanism and that anharmonicity or intrinsic phonon effects are relatively small. The PLQY trend with shell thickness in HgSe/CdS displays a fair agreement with a generic quartic fit, shown in Figure 5A, and the deviations at thicker shells are possibly due to irregular shell growth or strain defects. While the rate of surface trapping should be slow at the shell thicknesses considered here due to the exponential dependence on the tunneling barrier, it is possible that stacking faults in CdS arising from lattice strain can allow the carriers to reach the surface.^79^ We empirically observe that the intraband PLQY is relatively insensitive to the surface ligand coverage. Although this suggests that surface trapping is not significant, we cannot rule out a trapping mechanism with the current data.

It is generally reported that the interband emission of thick shell CdSe/CdS is dimer than for intermediate shells, possibly due to defects in the thick CdS shell. It is known that bulk crystalline CdS are photoresponsive in the near- and shortwave-infrared via absorption associated with deep traps,^72,73^ and the photoresponse can depend sensitively on the CdS growth conditions.^74^ Such traps in CdS might also have a negative impact on the photoluminescence quantum yields of both interband and intraband transitions of the HgSe core. For example, electron trapping by states in the shell could prevent bright interband and intraband emission (Figure S3F). Further progress in intraband emission will likely benefit from improved shell growth or a focus on shell materials that are defect-free.

The 2 ± 1% intraband PLQY achieved with thick-shell HgSe/CdS QDs makes them the brightest reported solution-phase chromophores in the 2000 cm^–1^ (5 μm) region at room temperature. This PLQY is also close to the room-temperature record of ∼4% observed in epitaxial III–V superlattices.^80,81^ The ability to engineer QDs with slow nonradiative relaxation is important for mid-infrared photodetectors, where background noise is fundamentally limited by the nonradiative relaxation of thermal carriers.^8^ Long lifetimes and high quantum yields should also support mid-infrared emission or lasing by enabling longer gain lifetimes, lower oscillation thresholds, and smaller saturation intensities.^82^ Our results indicate that decoupling the QD excitation from the infrared absorbing environment remains crucial for achieving long intraband lifetimes.

## Conclusions

Infrared nonradiative decay is ubiquitous in solution phase chromophores, and inorganic colloidal quantum dots provide an avenue toward brighter emitters. Here we focused on the intraband chromophores provided by n-doped HgSe quantum dots emitting at 5 μm. To obtain brighter photoluminescence and test the fundamental limitations imposed by phonon relaxation, we synthesized thick CdS shells on HgSe QDs. Control of the CdS growth in the zincblende crystal structure allows formation of compact shells up to thicknesses exceeding 7 nm, with total nanocrystal sizes approaching 20 nm. Although the doping disappears upon shell growth, we developed a procedure to regain and control the doping by a postsynthetic treatment with cadmium acetate. The photoluminescence quantum yields were then studied as functions of doping and shell thickness. At low n-doping levels, the HgSe/CdS QDs display the highest intraband PL quantum yields, up to 2% for the thickest shells, corresponding to intraband nonradiative lifetimes estimated in excess of 10 ns. Such lifetimes suggest that phonon-mediated relaxation is at least an order of magnitude slower than predicted by semiclassical electron–phonon relaxation calculations.^24,25^ The quantum yields reported in this study are the largest of all colloidal nanomaterials, interband or intraband, at 5 μm.

## Supporting Information Available

Protocols for syntheses of thin and thick shell HgSe/CdS QDs, n-doping of QDs, measurement of doping and PLQY, lifetime measurements, variations in thick shell HgSe/CdS synthesis, kinetics of Cd(PDTC)_2_ decomposition, calculations of dissolved fraction of thin shell HgSe/CdS QDs, radiative lifetimes, and population distribution of doped QDs (PDF)
