# Supplementary material for: Toward Bright Mid-Infrared Emitters: Thick-Shell n-Type HgSe/CdS Nanocrystals
Source: J Am Chem Soc. 2021 Nov 9;143(46):19567–75. doi: 10.1021/jacs.1c09858 (PMC8630792; doi:10.1021/jacs.1c09858)
Supplement: Supplementary file 1 — ja1c09858_si_001.pdf [file ja1c09858_si_001.pdf]

Supporting Information for Towards Bright Mid-Infrared Emitters: Thick-Shell n-Type HgSe/CdS Nanocrystals

Ananth Kamath, Christopher Melnychuk and Philippe Guyot-Sionnest*

Department of Chemistry and the James Franck Institute, The University of Chicago, 929 East 57th Street, Chicago, Illinois 60637, United States

*Corresponding Author Email: pgs@uchicago.edu

Table of contents

Section 1: Syntheses and surface modifications

1A: Chemicals

1B: Precursors and stock solutions

1C: HgSe core QD synthesis

1D: Thin shell HgSe/CdS QD synthesis by cALD

1E: Thin shell HgSe/CdS QD synthesis using $Cd(PDTC)_2$

1F: Testing the thermal stability of thin shell HgSe/CdS QDs

1G: Thick shell HgSe/CdS QD synthesis

1H: Doping control in HgSe/CdS QDs

1I: Variations in synthesis of thick shell HgSe/CdS QDs

1J: Kinetics of decomposition of $Cd(PDTC)_2$

Section 2: Characterizations

2A: Infrared absorption and photoluminescence measurements

2B: Photoluminescence lifetime measurements

2C: Measurement of PLQY and $1S_e$ occupancy

2D: Particle size characterizations

2E: Doping - dependent photoluminescence lifetime measurements

Section 3: Calculations and analyses

3A: Dissolution of thin shell HgSe/CdS QDs and deposition of HgCdSSe shell

3B: Radiative lifetime of HgSe and HgSe/CdS QDs

3C: Measurement of average diameter of bullet-shaped HgSe/CdS QDs

3D: Calculating the distribution of $1S_e(0)$, $1S_e(1)$ and $1S_e(2)$ QDs

3E: Fitting of intraband and interband absorptions

3F: Defect-assisted nonradiative relaxation in thick shell HgSe/CdS QDs

Section 4: References

1. Syntheses and surface modifications

1A: Chemicals

Ammonium hydroxide (NH_4OH, 28-30% in water), carbon disulfide (CS_2, 99.9%), cadmium chloride ($CdCl_2$, 99.99%), cadmium oxide (CdO, 99.9%), oleic acid (90%), cadmium nitrate tetrahydrate (99.997%), 1-dodecanethiol (DDT, 98%), oleylamine (OAm, 70%), hexadecane (99%), dodecylamine (98%), hexadecylamine (90%), 1-octadecene (ODE, 90%), cadmium acetate hydrate ($Cd(OAc)_2$, 99.99%), iodine (I_2, 99.99%), tetrachloroethylene (TCE, 99%) and ammonium sulfide ($(NH_4)_2S$, 40-40% in water) were purchased from Sigma-Aldrich. Mercury (II) chloride ($HgCl_2$, 99.999%), aniline (99%), didodecyldimethylammonium bromide (DDAB, 98%) and formamide (FA, 99%) were purchased from Alfa Aesar. Isopropanol (IPA, 99.9%) was purchased from Fisher, methyl acetate (99%) was purchased from Acros Organics, cadmium bis(diethyldithiocarbamate) ($Cd(DEDTC)_2$) (96%) was purchased from Gelest, and bis(trimethylsilyl)selenide (TMSSe) was purchased from Wonik Materials North America. All chemicals were used without further purification.

1B: Precursors and stock solutions

Synthesis of $Cd(PDTC)_2$: Cadmium bis(phenyldithiocarbamate) was synthesized following reported literature with minor changes.[1,2] For synthesis of the precursor ammonium phenyldithiocarbamate (NH_4PDTC),[1] 50 mL NH_4OH, 50 mL acetone and 20.1 mL aniline (220 mmol) were added to a 3-neck flask under nitrogen bubbling at room temperature. 13.3 mL CS_2 (220 mmol) was added dropwise with vigorous stirring. The solution developed a red color after 5 minutes, and a pale-yellow crystalline product slowly crystallized and precipitated over the next 90 minutes. The product was collected by vacuum filtration and washed with 120 mL cold ethanol. The NH_4PDTC was dried under vacuum for 3 hours and stored in a freezer to prevent decomposition. 11.2 g of product was obtained.

For synthesis of $Cd(PDTC)_2$,[2] 1.25 g NH_4PDTC was dissolved in 100 mL water in an Erlenmeyer flask. The solution was partially cloudy. 615 mg $CdCl_2$ was dissolved in 80 mL water and added dropwise to the flask over 30 minutes, leading to immediate precipitation of $Cd(PDTC)_2$ as a pale yellow powder. The product was collected by vacuum filtration, and

washed with 25 mL cold ethanol. The precipitate was ground with a mortar and pestle, dried under a Schlenk line and stored in a dessicator.

Synthesis of $Cd(oleate)_2$: Cadmium oleate was synthesized using a reported procedure.[3] It was necessary to ensure that the cadmium oleate contained no free oleic acid (as characterized by FTIR), as this resulted in QDs with poor intraband PLQY.

Synthesis of $Cd(DDT)_2$: Cadmium dodecanethiolate was synthesized adapting reported literature.[4] 1.55 g of cadmium nitrate (5 mmol) was dissolved in 25 ml methanol. In an Erlenmeyer flask, 12 mL triethylamine, 12 mL methanol and 2.01 g dodecanethiol were added. The cadmium nitrate solution was added to the flask dropwise with stirring to obtain a white precipitate. The product was collected by vacuum filtration, and washed three times with methanol. The precipitate was ground with a mortar and pestle, dried in a Schlenk line vacuum, and then stored under ambient conditions.

$Cd(PDTC)_2$ solution: A 0.1M solution of cadmium bis(phenyldithiocarbamate) in 20% OAm-hexadecane was prepared by adding 134mg (0.3mmol) of $Cd(PDTC)_2$ to 0.6mL oleylamine and 2.4mL hexadecane. The mixture was vortexed for 15 minutes at room temperature to dissolve.

Saturated amine solution: Prepared by mixing a 2:2:1 mass ratio of hexadecane-dodecylamine-hexadecylamine. The mixture was designed from the report by X Peng and coworkers,[5] but using longer chain molecules to increase the boiling point. The mixture was solid at room temperature, and had to be melted by warming to ~40°C before use.

20% oleylamine-hexadecane solution: 2 mL of oleylamine was mixed with 8 mL hexadecane.

Cadmium oleate solution: A 0.024M solution was prepared by adding 162mg (0.24mmol) of $Cd(oleate)_2$ to 10mL of saturated amine solution. The solution was vortexed at room temperature till the $Cd(oleate)_2$ fully dissolved.

Cadmium dodecanethiolate solution: A 0.024M solution was prepared by adding 123mg (0.24mmol) of $Cd(DDT)_2$ to 10mL of saturated amine solution. The solution was heated in air to ~100°C till the $Cd(DDT)_2$ fully dissolved. The reagent formed a fine gel-like precipitate

on cooling, but the precipitated powder was small enough to measure the reagent quantitatively.

$Cd(DEDTC)_2$ precursor solution: A 0.02M CdS precursor solution was prepared by adding 2mL of 0.024M $Cd(oleate)_2$ solution (or $Cd(DDT)_2$ solution) to 10mL of 0.024M cadmium bis(diethyldithiocarbamate) solution (98mg $Cd(DEDTC)_2$ in 10mL saturated amine solution). The mixture was sonicated at room temperature to dissolve, and was warmed to ~40°C before loading to a syringe pump.

$Cd(acetate)_2$ doping solution: A 0.05M solution of cadmium acetate was prepared by adding 13mg (0.05mmol) cadmium acetate dihydrate to 0.9mL hexadecane and 0.1mL oleylamine in a test tube. The mixture was heated in air to around ~100°C to dissolve the cadmium acetate. The solution formed a gel on cooling, but it could be dissolved on gentle warming.

I_2 solution for doping modification: A 0.02M solution of I_2 in TCE was prepared by adding 10mg I_2 to 2mL TCE and sonicating for 15 mins in a glass vial. A 0.004M solution was prepared by adding 0.4mL of this solution to 1.6mL TCE in another vial.

1C: HgSe core QD synthesis

HgSe core QDs were synthesized following the procedure by Melnychuk et. al.[6] Briefly, 108mg of $HgCl_2$ was added to a 3-neck flask with 10mL oleylamine. The flask was equipped with a stir bar, rubber sleeve stoppers with a thermocouple attached, and connected to a Schlenk line manifold. Three vacuum (p~1 torr) – Argon flush cycles were performed at room temperature, and the flask was heated at 100^0C for 45 minutes. The temperature was then set to 90^0C. A TMSSe solution (50uL in 0.95mL oleylamine) was prepared in a N_2-purged glovebox and injected swiftly into the flask. The temperature dropped to 88^0C and was kept at this temperature for 2 minutes. The flask was then cooled to room temperature using compressed air, and 10mL octane was added.

Purification of the QDs was done in air. On cooling, the solution was centrifuged to obtain the excess unreacted Hg- complex as a grey pellet. The supernatant containing the HgSe QDs was collected and precipitated by addition of ethanol, and centrifuged. The precipitate was

dispersed in 0.1mL oleylamine and 4mL TCE. The solution might appear foamy at this stage due to presence of excess Hg^{2+} species in solution. A second purification was done by addition of 0.8mL of 0.1M DDAB/TCE and precipitated with IPA. After centrifugation, the precipitate was dispersed in 0.1mL oleylamine and 2mL TCE and stored as a stock solution in the freezer. Concentration of the HgSe stock solution was determined by measuring the absorbance at 700 nm using the cross-section of HgSe as 3.5×10^{-18} cm^2 per Hg atom (calculated from the reported cross-section at 415 nm[6] and the measured HgSe absorption spectrum). Around 30mg of HgSe QDs were obtained from the reaction, giving a yield of ~50% with respect to the Selenium added.

1D: Thin shell HgSe/CdS QD synthesis by cALD

HgSe/CdS QDs were synthesized by cALD adapting the work by Shen et. al.[7] with notable changes. The reaction was done in a scintillation vial heated at 50-55°C, since this helped prevent the formation of an emulsion. No difference was observed between syntheses in air or in glovebox, so the reaction was done in air. The reaction was performed in dark to prevent photo-oxidation of sulfide to sulfates.

The $(NH_4)_2S$ stock solution was prepared by adding 1 mL of ammonium sulfide solution (40% in water) to 4 mL of formamide (FA) in the glovebox in a scintillation vial. The vial was capped with a rubber stopper and brought to the fume hood.

15mg of purified HgSe QDs was added to a scintillation vial with 4 mL of TCE, 0.4 mL OAm and equipped with a stir bar. 4 mL of formamide (FA) was added, and the vial was heated to 50-55°C with gentle stirring for 10 mins. The FA layer was discarded (to remove residual Hg^{2+}). For the first Cd layer, 4 mL FA was added with 30 mg cadmium acetate, and stirred at 50-55°C for 10 mins. The FA layer was then discarded, 4 mL FA was added and stirred for 2 mins. The FA layer was discarded to remove excess Cd^{2+} and the washing was performed a second time. The washings are an essential part of the procedure to prevent independent nucleation of CdS.

For the first S- layer, 4 mL FA was added, and 0.5 mL of the $(NH_4)_2S$ stock solution was added dropwise. The vial was stirred at 50-55°C for 2 mins. The FA layer was then discarded, 4 mL FA was added and stirred for 2 mins, and then discarded. The washing was done for a second

time. Three more Cd- and S- layers were similarly performed using 32 mg, 35 mg and 40 mg cadmium acetate respectively, and 0.5 mL $(NH_4)_2S$ stock solution. A fifth Cd- layer using 45 mg cadmium acetate was performed to n-dope the QDs. The stirring was done gently to avoid formation of an emulsion. If an emulsion was formed, few drops of isopropanol (IPA) were added to aid in the separation of the layers.

After the synthesis of the thin CdS shell, the QDs were purified twice by precipitation – dispersion using IPA and TCE.

It is important to note that the cALD reaction is sensitive to the stirring speed. Excessive stirring leads to formation of an emulsion and incomplete removal of excess Cd^{2+} and S^{2-}, leading to independent nucleation of CdS.

1E: Thin shell HgSe/CdS QD synthesis using $Cd(PDTC)_2$

Unless otherwise noted, all syntheses of thin shell HgSe/CdS QDs were performed using this method. Cadmium bis(phenyldithiocarbamate) ($Cd(PDTC)_2$) was used as highly reactive single-source precursor for deposition of a thin shell on HgSe at low temperatures, motivated by the work by Buhro and coworkers.[2]

30mg of purified HgSe core QDs was added to a 3-neck flask, and 15mL of a 20% oleylamine – hexadecane solution was added. Three vacuum (p ~ 1 torr) – argon evacuation cycles were performed at room temperature. The temperature was then set to 80°C. When the temperature reached 40°C, 1.17mL of 0.1M $Cd(PDTC)_2$ solution (2 monolayers (ML) of CdS) was injected. The solution was kept at 80^0C for 5 minutes. The flask was then cooled to 50°C and 0.82mL of 0.1M $Cd(PDTC)_2$ solution (1 ML of CdS) was injected, and the solution was heated at 80°C for 5 minutes. The flask was then connected to vacuum at 80°C to remove the H_2S formed during the shell growth. When the pressure dropped below 1 torr, the flask was returned to Argon and cooled to room temperature.

The HgSe/CdS QDs showed more colloidal stability than the HgSe cores, and did not require the addition of DDAB during precipitations. The HgSe/CdS QDs were purified in air by precipitation using methyl acetate. Some oily residue was observed after synthesis which is difficult to remove using precipitation / dissolution. 2 mL TCE was added to the black

precipitate, and the solution was stored as a stock solution in a vial in the freezer. TEM imaging showed that the QD diameter increased from 4.8 nm to 6.1 nm after the shell growth. No homogenous nucleation was observed at the above reaction conditions, though homogenous nucleation was seen when the $Cd(PDTC)_2$ precursor volume was doubled.

The concentration of the QDs was determined using the absorbance at 700nm using the cross-section of HgSe, under the assumption that the shell growth leads to negligible change in absorbance at this wavelength.

1F: Testing the thermal stability of thin shell HgSe/CdS QDs

Prior to the thick CdS shell synthesis, the thin shell HgSe/CdS QDs were annealed in solution to test for thermal stability. Thin shell HgSe/CdS QDs (containing 3 mg of HgSe cores) was purified by precipitation with methyl acetate. 1.5 mL of saturated amine solution was used to dissolve the QDs, and transferred to a 3-neck flask. 8.2mg $Cd(oleate)_2$ (1.5 surface equivalents) was then added. Three vacuum-Ar cycles were performed, and the solution was heated and kept at 220°C for a desired duration. The solution was cooled to room temperature and precipitated with methyl acetate. The pellet was dispersed in 0.9mL of TCE and 0.1mL oleylamine. The solution was heated to boil (~110°C - 120°C) for a few seconds to dissolve the saturated amines and passivate the QDs with oleylamine. After cooling to room temperature, the solution was purified twice by precipitation-dispersion using methyl acetate and TCE. The pellet after the second precipitation was dried under vacuum before dispersing in TCE for FTIR measurements. Apart from an increase in the n-doping, no observable change was seen in the thin HgSe/CdS QDs after annealing at 220°C (Fig. S1F).

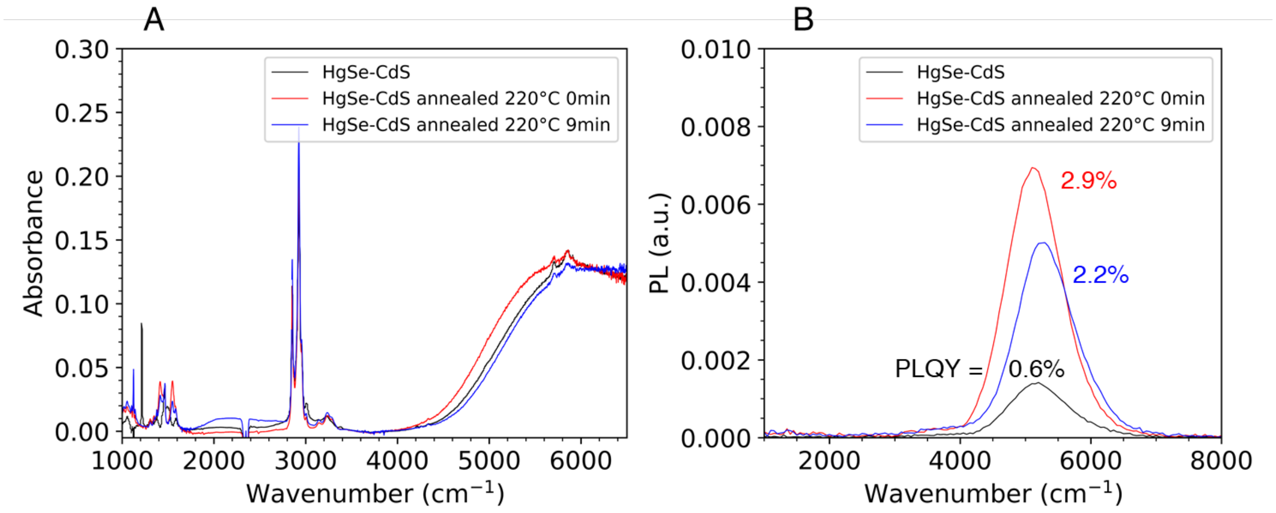

Fig. S1F: (A) Absorption spectra of thin shell HgSe/CdS QDs (diameter = 6.1 nm) before and after annealing in solution at 220°C with cadmium oleate. A small increase in the doping is observed due to the presence of cadmium oleate in solution, but the optical spectra are nearly unchanged, demonstrating the thermal stability of the thin shell HgSe/CdS QDs. (B) PL spectra of thin shell HgSe/CdS QDs before and after annealing at 220°C. Due to the low-temperature synthesis of the thin shell, the interband PLQY is relatively low at 0.6% (black), but rises to ~2-3% on annealing (red and blue). The intraband PL is too weak to be resolved at this scale.

1G: Thick shell HgSe/CdS QD synthesis

A thick CdS shell was overgrown on the thin shell HgSe/CdS QDs using $Cd(DEDTC)_2$ as a single-source precursor, with amines and $Cd(oleate)_2$ as ligands. The $Cd(oleate)_2$ helped prevent the growth of wurtzite arms during shell growth,[8,9] The concentration of $Cd(oleate)_2$ is crucial, as the sulfur-containing side products from decomposition of $Cd(DEDTC)_2$ reacted with excess $Cd(oleate)_2$ and led to undesired homogenous nucleation of CdS. We found that a 1:5 molar ratio of $Cd(oleate)_2$: $Cd(DEDTC)_2$ provided a balance to prevent growth of wurtzite arms, while avoiding homogenous nucleation. Cadmium dodecanethiolate ($Cd(DDT)_2$) was observed to give similar results as cadmium oleate when used as the ligand.

The synthesis was done in the absence of unsaturated solvents, as they led to the formation of a thick oil /gel after synthesis which was difficult to separate from the QDs. We hence employed a 2:2:1 mixture of hexadecane-dodecylamine-hexadecylamine as the solvent, where the mixture aided in entropic dispersion of the QDs.[10]

Thin shell HgSe/CdS QDs (containing 8mg of HgSe cores) was precipitated with methyl acetate to remove leftover oleylamine. The pellet was dispersed in 4mL of warmed saturated amine solution, and 1.35mL of 0.024M Cd(oleate)$_2$ solution (1.5 surface equivalents) was added. The mixture was added to a three-neck flask equipped with rubber sleeves and a thermocouple, and connected to a Schlenk line. The 0.02M Cd(DEDTC)$_2$ precursor solution was kept in a plastic syringe connected to a syringe pump. Three vacuum (p ~ 1 torr) - Argon cycles were performed, and the solution was heated rapidly to 220°C (average heating rate is around 50°C/min). When the temperature reached 215°C, the precursor solution was injected at rate 0.396 mL/min for 10 mins (equivalent to 3MLs of CdS). The temperature was maintained at 220°C and was not let to exceed 225°C. The flask developed a yellow tinge after a few minutes, indicating the growth of CdS. Depending on the reaction scale and heating rate, a reddish tinge might be observed, indicating dissolution of a fraction of the QDs and deposition of a HgCdSSe shell on the larger QDs.

After 10 mins, the injection was stopped and the syringe was removed. The solution was cooled to 120°C and evacuated to remove the H_2S formed during the reaction. Removal of the H_2S was necessary to prevent CdS homogenous nucleation during post-synthetic n-doping or during further CdS shell growth.

For a further growth of 3MLs of CdS, the flask was heated to 220°C and 0.02M CdS precursor injection was resumed at 0.650 mL/min for 10 mins (equivalent to 3MLs of CdS). The solution becomes cloudy with time as the dots grow larger. After the injection is complete, the flask was then cooled to 120°C and evacuated. The solution was then cooled to room temperature.

All purifications were done in air. On centrifuging the cooled reaction mixture, the nanocrystals typically precipitated as a thick bulky precipitate. Remaining QDs in the solution were precipitated using methyl acetate and centrifuged. After decanting the supernatant, the precipitate was dispersed in ~4mL TCE and 0.4mL OAm was added. The solution was brought to a boil (~110°C - 120°C) for a few seconds to dissolve the saturated amine solvent, and let to cool. The solution was then purified twice by precipitation with methyl acetate and dispersion in TCE. After the second precipitation, the pellet was dried under vacuum, and then dispersed in TCE for measurements.

The shape of the thick shell HgSe/CdS QDs was sensitive to the reaction scale, where a 4 mg reaction scale led to more compact QDs than an 8 mg scale.

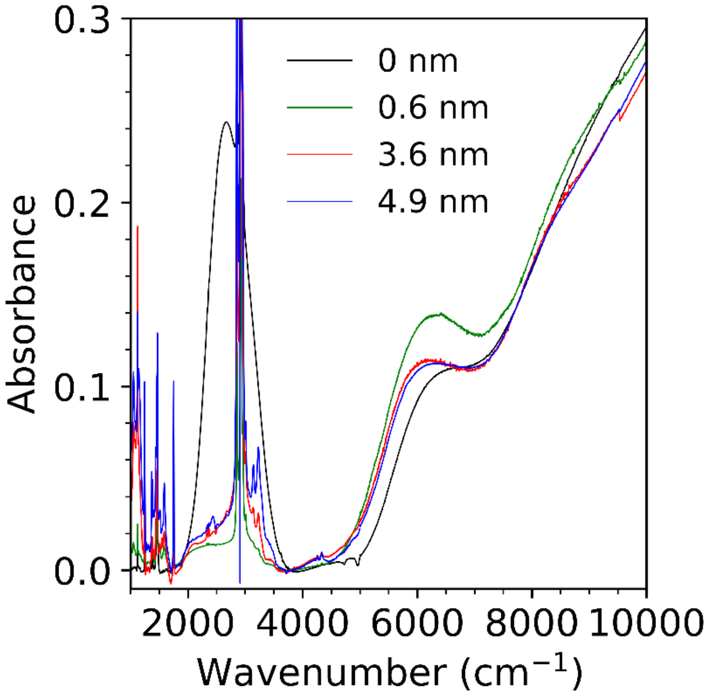

Fig. S1G: FTIR absorption spectra of HgSe QDs and HgSe/CdS QDs with different shell thicknesses (corresponding to samples in Fig. 1F in the main text). Negligible shifts in the intraband and interband absorptions of HgSe confirms that the integrity of the core is preserved after the thick shell growth. The data is obtained by stitching spectra using an FTIR spectrometer (<5000 cm^{-1}) and a dispersive NIR spectrophotometer (>5000 cm^{-1}).

1H: Doping control in HgSe/CdS QDs

The as-synthesized HgSe/CdS QDs typically showed a partial n-doping, as seen by the prominent $1S_h$ – $1S_e$ interband absorption and small $1S_e$ – $1P_e$ intraband absorption. To achieve doping control, we first completely undoped the QDs by performing a cALD with $(NH_4)_2S$, and then treated with cadmium acetate to achieve the desired n-doping level.[11] Reaction of cadmium acetate with thick shell HgSe/CdS QDs at room temperature led to incomplete n-doping, and it was necessary to perform the surface treatment at an elevated temperature to ensure a complete reaction.

The undoping was performed adapting a reported procedure.[7] At ambient conditions, purified 11nm HgSe/CdS QDs (core diameter 4.8nm, HgSe mass = 6mg) was added to a scintillation vial with 0.3 mL oleylamine and 2.7 mL octane. Separately, 0.16 mL of 40% $(NH_4)_2S$ solution (10x surface equivalents to HgSe/CdS) was mixed with 3mL formamide (FA), and added to the HgSe/CdS solution to form a biphasic mixture. The vial was heated at 40°C with stirring for 5 minutes. The FA layer was removed, and washed with 3 mL of FA to remove excess $(NH_4)_2S$. The washing was repeated another time, after which the QDs were purified twice by precipitation-dispersion using methyl acetate and TCE. After treatment with $(NH_4)_2S$, the intraband absorption was completely bleached (Fig. S1H).

To n-dope the QDs, the undoped HgSe/CdS QDs (containing 6 mg of HgSe cores) were added to a three-neck flask with 0.6 mL of oleylamine and 5.4 mL hexadecane. After adding a calculated volume of 0.05M cadmium acetate solution (see Fig. 2D of main text. 1.0 mL of cadmium acetate corresponded to 1x surface saturation), the flask was connected to a Schlenk line and three vacuum-Argon cycles were performed. The flask was then heated to 180°C and kept at this temperature for 10 mins.

The reaction mixture was cooled to room temperature, and purified twice by precipitation – dispersion using methyl acetate and TCE. After the second precipitation, the pellet was dried under vacuum before dispersing in TCE for measurements.

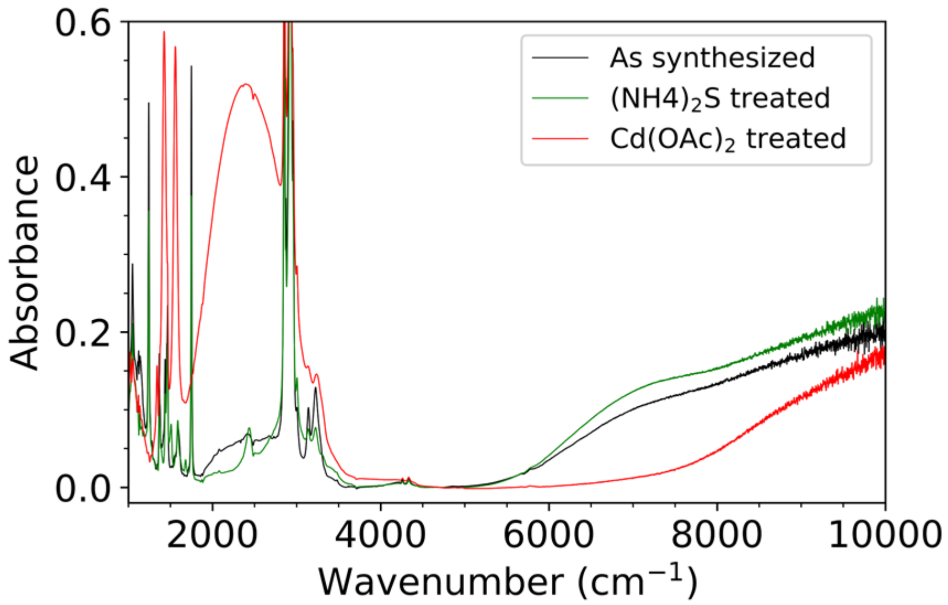

Fig. S1H: FTIR spectra of HgSe/CdS QDs (black) as synthesized, (green) after cALD with $(NH_4)_2S$ and (red) after treatment with cadmium acetate. The doping (N_e) could be controlled to any value between 0 and 2 electrons per QD.

1I: Variations in synthesis of thick shell HgSe/CdS QDs

Synthesis at 160°C with Cd(oleate)$_2$:

Reaction solvent was a 10% solution of OAm in ODE.

The CdS precursor solution (1.5 mg/mL CdS) was prepared by dissolving 46 mg Cd(DEDTC)$_2$ and 74 mg Cd(oleate)$_2$ in 20 mL 10% OAm-ODE by vortexing at room temperature.

Thin shell HgSe/CdS QDs (6 nm diameter, synthesized by cALD) containing 3.3 mg of HgSe cores were purified and added to a 3-neck flask with 2.5mL of 10% OAm-ODE, and 4mg of Cd(DEDTC)$_2$ (corresponding to 1ML of CdS) was added. The flask was evacuated at room temperature in a Schlenk line for 10 minutes, and then filled with Argon. The flask was then heated to 140°C and kept at this temperature for 1 hour. The flask was then heated to 160°C and the CdS precursor was injected using a syringe pump at 1.31 mL/hour. Aliquots were taken and purified twice by precipitation with IPA and dissolution in TCE for TEM imaging.

The shell grew like tetrahedra after 4 hours of injection (Fig. S1I(A)), but became irregular at 19 hours (Fig. S1I(B))

Synthesis at 220°C without $Cd(oleate)_2$:

Reaction solvent was the saturated amine mixture: a 2:2:1 mixture of hexadecane-dodecylamine-hexadecylamine by mass.

The CdS precursor solution (0.02M in CdS) was prepared by dissolving 164 mg $Cd(DEDTC)_2$ in 20 mL saturated amine mixture by vortexing at room temperature.

Thin shell HgSe/CdS QDs (6 nm diameter, synthesized by using $Cd(PDTC)_2$) containing 4 mg of HgSe cores were purified and added to a 3-neck flask with 2 mL of saturated amine mixture. Three vacuum – argon evacuation cycles were performed at room temperature, with evacuation till 1 torr. The flask was then heated to 220°C. When the temperature reached 200°C, the CdS precursor solution was injected at a rate 0.26 mL/min (3ML CdS in 10 mins). Aliquots were taken and purified by precipitation with IPA and dissolution in TCE for TEM imaging. The shell grew as rods after 10 mins of injection (Fig. S1I(C)), with arms developing at 25 mins (Fig. S1I(D)). This corresponds to a large (103) wurtzite feature in the SAED during transmission electron microscopy (Fig. S1I(K))

Synthesis at 220°C with $Cd(oleate)_2$:

Reaction solvent was the saturated amine mixture: a 2:2:1 mixture of hexadecane-dodecylamine-hexadecylamine by mass.

The CdS precursor solution (0.02M in CdS) was prepared by dissolving 164 mg $Cd(DEDTC)_2$ in 20 mL saturated amine mixture by vortexing at room temperature. A 0.1M solution of $Cd(oleate)_2$ was prepared by adding 17 mg $Cd(oleate)_2$ to 0.25mL of saturated amine mixture and vortexing at room temperature.

Thin shell HgSe/CdS QDs (6 nm diameter, synthesized by using $Cd(PDTC)_2$) containing 4 mg of HgSe cores were purified and added to a 3-neck flask with 2 mL of saturated amine mixture, and 0.14 mL of cadmium oleate solution was added. Three vacuum – argon evacuation cycles were performed at room temperature, with evacuation till 1 torr. The flask was then heated to 220°C. When the temperature reached 220°C, the CdS precursor solution

was injected at a rate 0.26 mL/min (3ML CdS in 10 mins). A 2.4 mL aliquot was taken at 10 mins, 0.05 mL of cadmium oleate was added and the CdS precursor was injected at 0.25 mL/min for 13 mins. The aliquots were purified twice by precipitation with IPA and dissolution in TCE for TEM imaging. The shell grew as uniform spheres at 10 mins of injection (Fig. S1I(E)), with some faceting visible at 23 mins (Fig. S1I(F)). The addition of cadmium oleate leads to a reduced (103) wurtzite feature in the SAED during transmission electron microscopy (Fig. S1I(K)).

Synthesis at 220°C with excess Cd(oleate)$_2$:

Reaction solvent was the saturated amine mixture: a 2:2:1 mixture of hexadecane-dodecylamine-hexadecylamine by mass.

The CdS precursor solution (0.02M in CdS) was prepared by dissolving 164 mg Cd(DEDTC)$_2$ in 20 mL saturated amine mixture by vortexing at room temperature. A 0.1M solution of Cd(oleate)$_2$ was prepared by adding 135 mg Cd(oleate)$_2$ to 2 mL of saturated amine mixture and vortexing at room temperature.

Thin shell HgSe/CdS QDs (6 nm diameter, synthesized by using Cd(PDTC)$_2$) containing 4 mg of HgSe cores were purified and added to a 3-neck flask with 2 mL of saturated amine mixture, and 0.54 mL of cadmium oleate solution was added. Three vacuum – argon evacuation cycles were performed at room temperature, with evacuation till 1 torr. The flask was then heated to 220°C. When the temperature reached 220°C, the CdS precursor solution was injected at a rate 0.27 mL/min (3ML CdS in 10 mins). After 10 mins, the solution was cooled to room temperature, divided into two parts and precipitated with IPA. One part was purified twice by precipitation – dispersion using IPA and TCE for TEM imaging. The other part was precipitated once with IPA, dispersed in 2.6 mL saturated amine mixture, and moved to the 3-neck flask with 0.55 mL of 0.1M cadmium oleate. After three vacuum – Argon cycles, the flask was heated to 220°C and the CdS precursor solution was injected at 0.139 mL/min for 10 mins. The solution was purified twice and imaged by TEM.

The QDs grew as uniform spheres till 10 mins (Fig. S1I(G)), but homogenous nucleation of CdS was observed at 20 mins (Fig. S1I(H)).

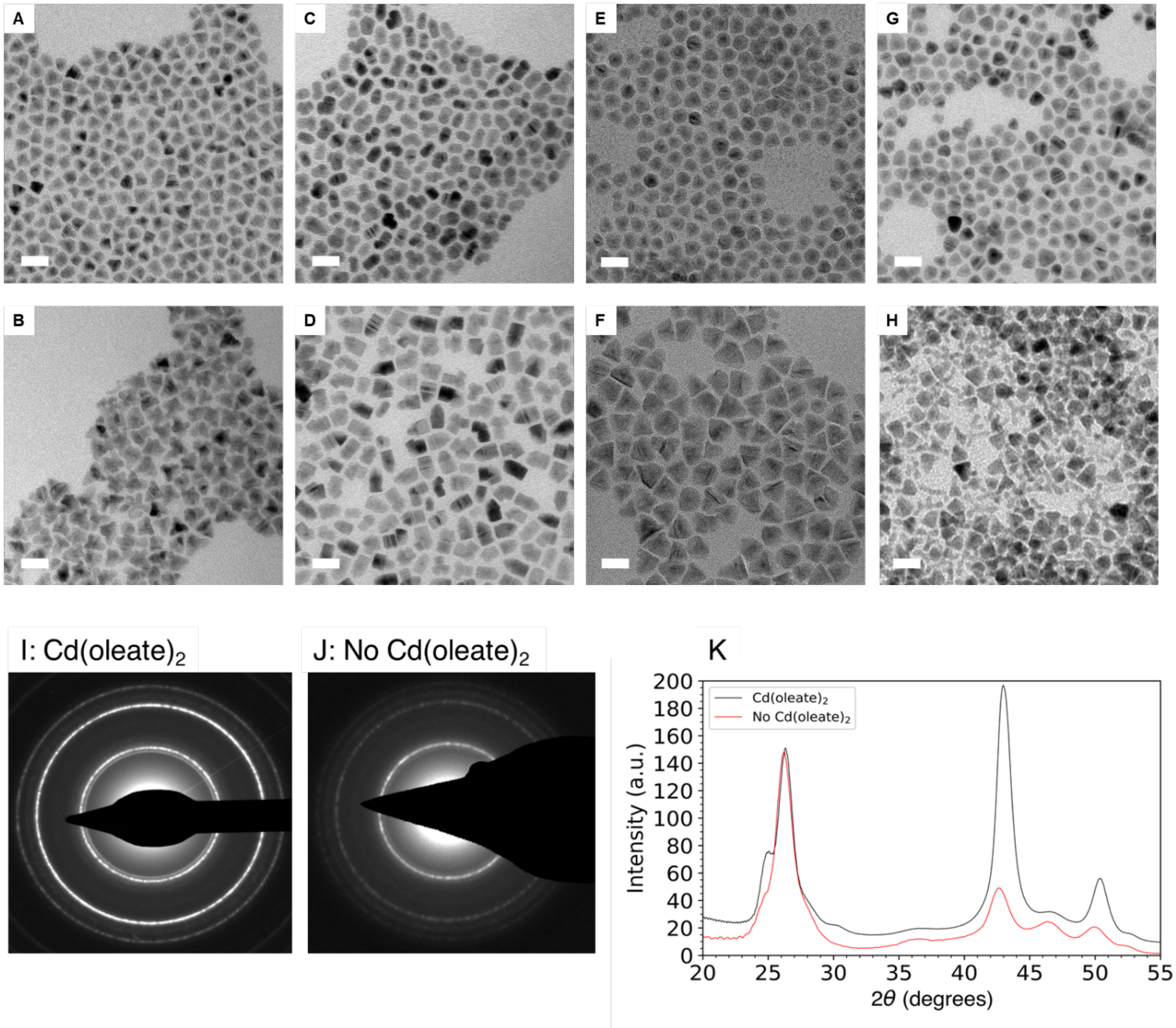

Fig. S1I: TEM images of thick shell HgSe/CdS QDs synthesized by different methods: (A, B) at 160°C with cadmium oleate, (C, D) at 220°C without cadmium oleate, (E, F) at 220°C with ~1.5 surface equivalents of cadmium oleate, (G, H) at 220°C with excess cadmium oleate. All scale bars are 20 nm. (I, J) SAED images of HgSe/CdS QDs synthesized at 220°C (I) with cadmium oleate and (J) without cadmium oleate. The samples correspond to TEM images in (D) and (F) respectively; (K) radial integration of the SAED spectra to calculate the pXRD spectra. Addition of cadmium oleate during the reaction leads to a reduced (103) wurtzite feature in the pXRD (at 47°) and more compact shell growth.

1J: Kinetics of decomposition of $Cd(PDTC)_2$

To determine to temperature for growth of a thin CdS shell on HgSe using $Cd(PDTC)_2$, we performed a kinetics study on the decomposition of $Cd(PDTC)_2$. A solution of 0.1 M $Cd(PDTC)_2$ in 20% OAm-ODE was prepared.

Thin HgSe/CdS QDs (synthesized using $Cd(PDTC)_2$) containing 4 mg of HgSe was added to a 3-neck flask with 2 mL of 20% OAm-ODE solution. Three vacuum-argon cycles were performed, and the solution was heated to 60°C. 0.33mL of $Cd(PDTC)_2$ solution was injected. Aliquots were taken immediately (0 mins), 2 mins, 5 mins, 10 mins and 30 mins after injection. The aliquots were stored in a freezer to prevent further reaction.

Absorption spectra of the aliquots were recorded in a quartz cuvette by dilution in TCE, and normalized to absorbance at 1000 nm. The kinetics study was also performed for reaction at 80°C. As shown in Fig. S1J, the reaction is nearly complete in less than 2 mins at 80°C but took 10 mins at 60°C. The reaction temperature for growth of thin shell HgSe/CdS QDs using $Cd(PDTC)_2$ was thus set at 80°C.

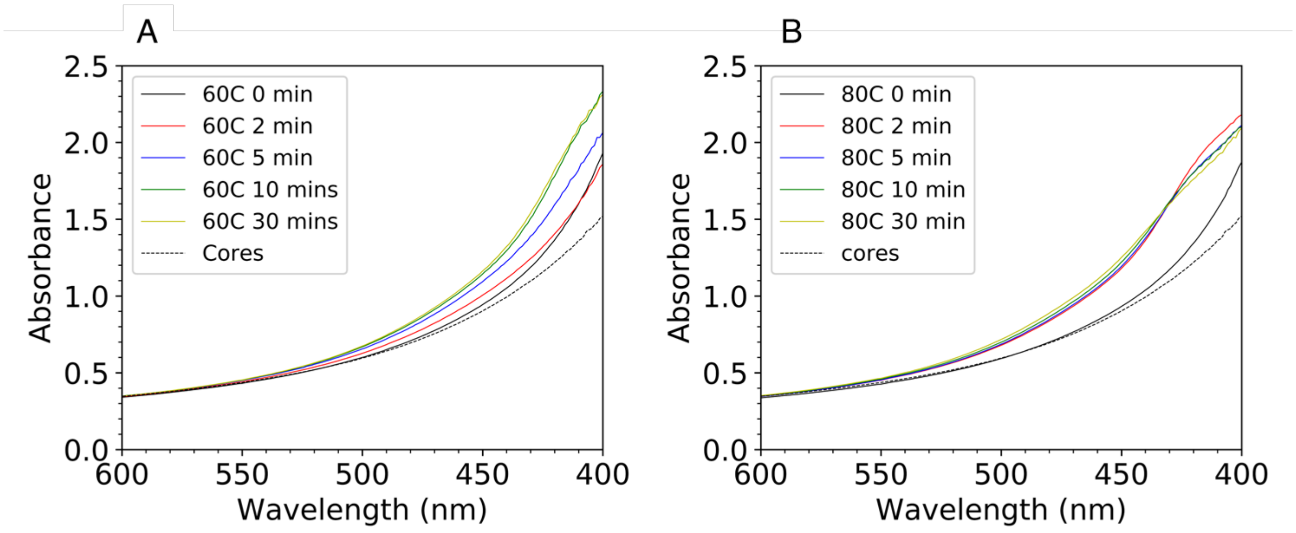

Fig. S1J: Absorption spectra of thin shell HgSe/CdS QDs after injection of $Cd(PDTC)_2$ at (A) 60°C or (B) 80°C, at different times after the injection. The reaction takes 10 mins to complete at 60°C, but finishes in less than 2 mins at 80°C. All spectra are normalized to absorbance at 1000 nm.

2. Characterizations

2A: Infrared absorption and photoluminescence measurements

All measurements were done on QDs dispersed in TCE in a cell with CaF_2 windows and a path length of 0.5mm. Absorption measurements were performed on a ThermoNicolet Nexus 670 FTIR spectrometer, a Nicolet Magna 550 FTIR spectrometer and an Agilent Cary 5000 UV-vis-NIR spectrometer.

Photoluminescence spectra were recorded using a step-scan FTIR spectrometer with an MCT detector and a gated integrator. The samples were excited with a 150mW 808 nm diode laser, modulated at 90 kHz. A Si wafer was placed in front of the detector to block the excitation light. The transmittance of the solution at 808nm was measured using a Si diode detector behind the sample cell. A 3.4µm long-pass filter was placed in front of the detector to block the 1.7 µm interband emission and preferentially measure the 5 µm intraband emission. The PL spectrum was corrected for detector response and atmospheric absorption following Melnychuk et. al.[6] The PL spectrum was normalized by the measured 808 nm absorbance, and was also corrected for the infrared absorption by TCE.

2B: Photoluminescence lifetime measurements

Photoluminescence lifetime measurements were performed using the upconversion apparatus described by Melnychuk et. al.[6] with minor modifications. Samples were dispersed in TCE and placed in a homemade CaF_2 cuvette for the measurements. The beam from a home-built Nd:YLF regenerative amplifier producing 6 ps, 1053 nm pulses at 1 kHz was split into two paths by an 80:20 beamsplitter. The weak path pumped the sample after attenuation, variable optical delay, and weak focusing by a spherical lens to a spot size of 0.5 mm at the sample. The pump optical fluence at the sample was 150 $\mu J/cm^2$. The strong path was brought collinear with the intraband fluorescence by a homemade CaF_2 beamsplitter and parametric mixing in a KTA crystal generated light at the sum-frequency. Filters removed the fundamental and weak second harmonic of the laser. The sum-frequency pulse then passed through a monochromator to a silicon photomultiplier and a gated integrator discriminated it from the background. The spectral resolution of the measurement at 2050

cm^{-1} was 70 cm^{-1} FWHM. Due to the large optical density at 1053 nm of the samples, emission from only a thin liquid layer is detected and the time resolution is expected to be about 8 ps.

2C: Measurement of PLQY and $1S_e$ occupancy

Absolute PL quantum efficiency measurements (PLQE) were done following previous reports[6] using a Spectralon integrating sphere for a reference partially doped HgSe/CdS QD solution. The area under the PL spectrum of HgSe/CdS QDs (normalized to 808 nm absorption and corrected for detector response) was taken between 1600 – 2800 cm^{-1} for intraband, and 4000 – 7000 cm^{-1} for interband spectra. The ranges were taken as 1600 – 3000 cm^{-1} and 5000 – 8000 cm^{-1} for the HgSe cores. The PLQE per unit area was then calculated by dividing the measured PLQE by sum of areas under the intraband and interband PL spectra. The PLQE of other samples were calculated by recording the PL spectra and using the PLQE per area calculated above.

The average occupancy of the $1S_e$ state (N_e) in the HgSe/CdS QDs was determined by undoping the QDs on treatment with I_2. FTIR absorption spectra of the QDs in TCE were measured before and after injection of I_2 in TCE (0.02M or 0.004M). Difference between subsequent spectra were used to calculate the $1S_e$ – $1P_e$ intraband absorption (see Fig. S2C-1 for details).

N_e was then calculated as twice the ratio of intraband peak absorbance to the maximum intraband absorbance (Y-intercept in Fig. 2(C) in main text). The $1S_e(0)$, $1S_e(1)$ and $1S_e(2)$ populations were calculated from N_e using a binomial distribution (SI Section 3D). The fraction of doped QDs was taken as sum of $1S_e(1)$ and $1S_e(2)$ populations, and undoped fraction was $1S_e(0)$. The intraband and interband PLQYs were calculated by normalizing the measured PLQEs to the fraction of doped and undoped QDs respectively.

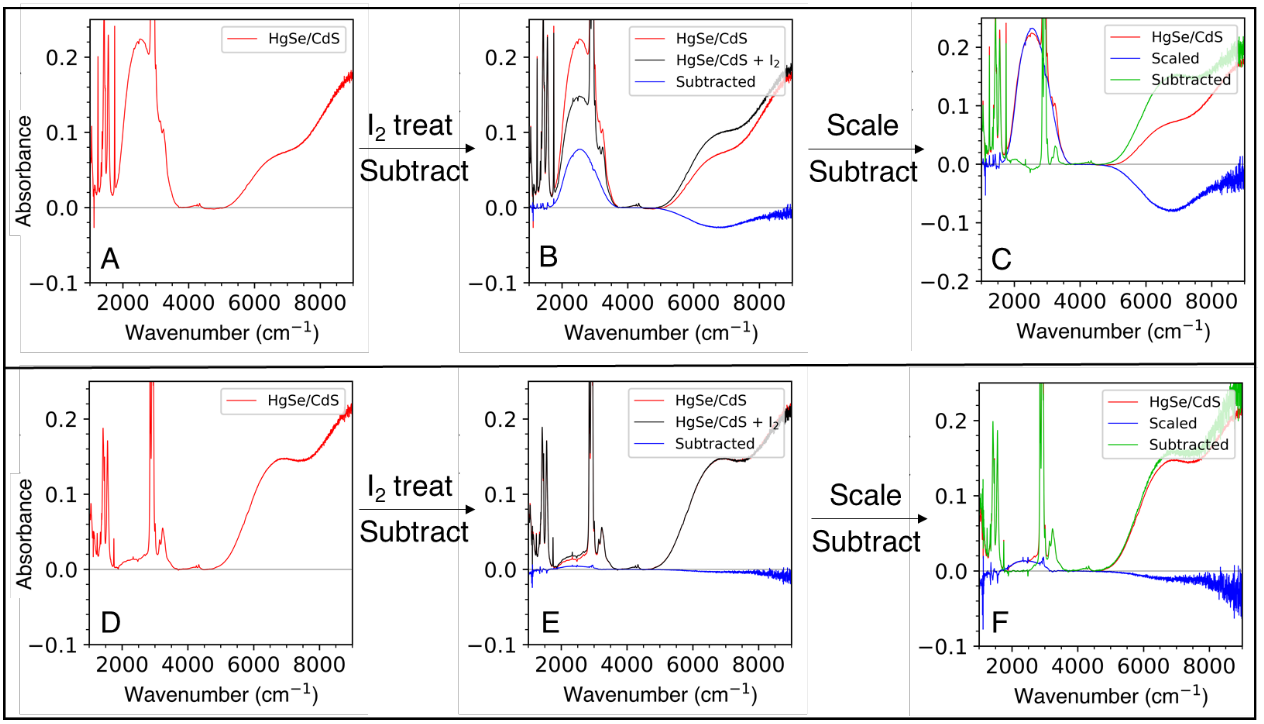

Fig. S2C-1: Subtraction to measure the intraband absorbance for HgSe/CdS QDs with (A) high doping (N_e = 1.2) and (D) low doping (N_e = 0.07). (B, E): The sample is treated with I_2 / TCE solution to change the doping (black spectra). The doping decreases if the initial doping was (B) high, and increases if it was (E) low. A subtraction was performed to remove the ligand absorption (blue spectra), resulting in a spectrum with intraband absorption and interband bleach. (C, F): The subtracted spectra from (B) and (E) are then scaled (blue spectra) to match the intraband absorbance of the original HgSe/CdS QDs. Taking a difference between the HgSe/CdS and the scaled spectra gives a sum of ligand and interband absorbance (green spectra). Despite the low n-doping in (D), the subtraction procedure can quantitatively give the intraband absorption (see Fig. S2C-2).

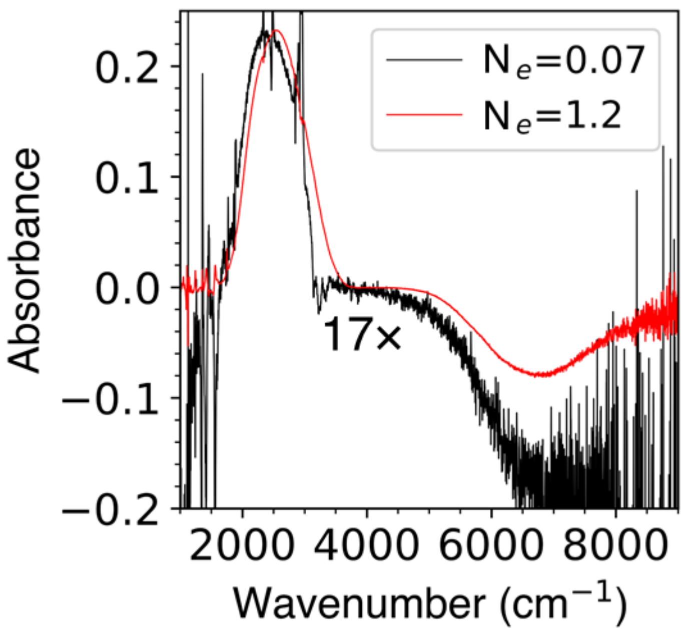

Fig. S2C-2: Intraband absorbances of HgSe/CdS QDs from Fig. S2C-1, scaled to compare the lineshapes. The intraband shapes match well, showing the robustness of the subtraction procedure. The interband shape of the $N_e = 0.07$ sample deviates significantly due to scattering artefacts during FTIR measurements.

2D: Particle Size Characterizations

Transmission Electron Microscopy (TEM) images were recorded using an FEI Spirit 120kV electron microscope and an FEI Tecnai F30 300kV microscope. pXRD spectra were recorded on QDs films on Si using a Bruker D8 Powder X-Ray Diffractometer.

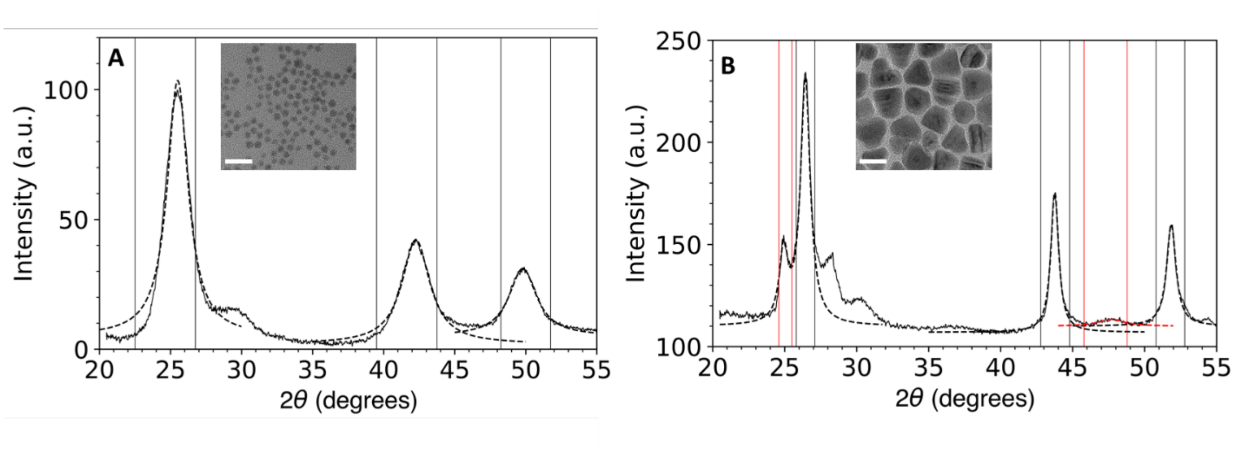

Fig. S2D: Scherrer analysis and Lorentzian fittings (dotted curves) for pXRD of (A) 4.8 nm HgSe and (B) 19 nm HgSe/CdS QDs. TEM images are shown in the insets, with a scale bar of 20 nm. Vertical black lines are the ranges used for the fittings of zincblende peaks, and the vertical red lines is the range used for fitting the (100) and (103) wurtzite peaks. The Scherrer sizes obtained are (4.0 ± 0.5) nm for HgSe, and (11.7 ± 1.5) nm for HgSe/CdS. The smaller Scherrer crystalline size compared to the particle size on TEM is likely due to the presence of grain boundaries and defects in the nanocrystals. To estimate the Wurtzite contribution in the HgSe/CdS spectrum, we assumed that the (100), (002) and (101) peaks have equal intensity, and the peak at 26° includes intensities from wurtzite (002) and zincblende (111) peaks. The zincblende:wurtzite ratio is then calculated as ratio of the areas of the zincblende (111) and wurtzite (100) peaks, which is found to be 3:1. Ratio of average area under the zincblende (220) and (311) peaks with area under the wurtzite (103) peak is 5:1.

2E: Doping – dependent transient lifetime measurements

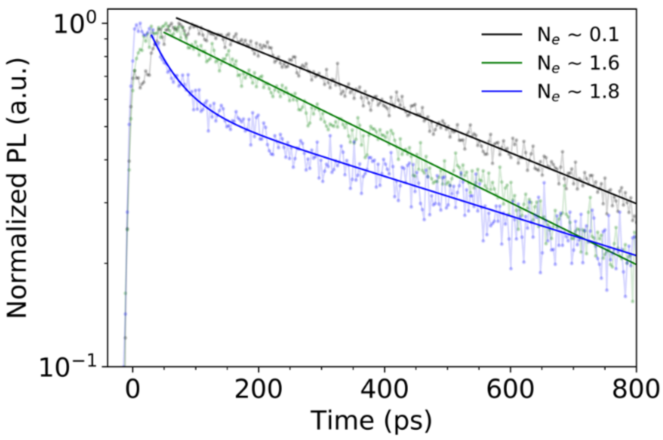

$$y(t) = ae^{-bt} + ce^{-dt}$$

N_e	a	b (ps)	c	d (ps)
0.1	1.165 ± 0	587 ± 26	-	-
1.6	1.042 ± 0	482 ± 23	-	-
1.8	0.609 ± 0	750 ± 540	0.660 ± 0.003	44 ± 18

Fig. S2E: Normalized transient PL measurements (points) on 12 nm HgSe/CdS QDs with different $1S_e$ occupancies (N_e) by doping with cadmium acetate. The $N_e \sim 0.1$ and $N_e \sim 1.6$ were fit to single exponentials, while the $N_e \sim 1.8$ was fit to a biexponential. The fitting parameters show that the average lifetime reduces with increase in doping. The noise baseline of the transient PL measurement is at 0.01. The features in early time dynamics suggest that intersublevel relaxation might depend on the doping level.[6]

3. Calculations and analyses

3A: Dissolution of thin shell HgSe/CdS QDs and deposition of HgCdSSe shell

During the initial stages of the thick shell HgSe/CdS synthesis, a fraction of the thin shell HgSe/CdS QDs dissolve and deposit on the remaining QDs as a HgCdSSe shell. This leads to a red tail in the visible spectrum beyond the CdS band edge, and larger core/shell sizes than calculated from the precursors added. A batch-to-batch variability is observed in the extent of dissolution, and it also depends on the heating rate. Two synthetic trials are described below that show a substantial QD dissolution (synthesis 1) and a negligible dissolution (synthesis 2).

Synthesis 1 and 2 were performed by the thick shell HgSe/CdS synthesis procedure mentioned in the **Methods** section. The reaction conditions were nearly identical, except that the syntheses used different batches of thin shell HgSe/CdS QDs, and synthesis 1 used started with a scale of 18 mg of HgSe, while synthesis 2 started with a scale of 6 mg of HgSe.

As shown in Fig. S3A(A, B), the core/shell sizes in synthesis 1 match well with the diameter expected from the $Cd(DEDTC)_2$ precursor added, but the synthesis 2 leads to much larger core/shell diameters. From the size discrepancy in synthesis 2, the fraction of dissolved QDs can be calculated:

Calculation of fraction of dissolved QDs

Denote the initial diameter as d_i , the expected final diameter as d_{exp} , measured final diameter as d_m, and initial number of nanocrystals as N. The initial volume of QDs is $N\frac{\pi}{6}d_i^3$, volume of shell material added is $N\frac{\pi}{6}(d_{exp}^3 - d_i^3)$. The total volume of QD material (HgSe + CdS) is then $N\frac{\pi}{6}(d_{exp}^3)$. Suppose a fraction x of thin shell HgSe/CdS QDs dissolve during initial stages of the thick shell synthesis. Now the volume of core HgSe material remaining is $N\frac{\pi}{6}d_i^3(1-x)$, volume of HgCdSSe shell material is $N\frac{\pi}{6}(d_{exp}^3 - d_i^3(1-x))$. Since the final diameter of the QDs is: $Initial\ QD\ diameter \times \left(\frac{Total\ QD\ volume}{Initial\ QD\ volume}\right)^{\frac{1}{3}}$, we have

$$d_f = d_i \times \left(\frac{N\frac{\pi}{6}(d_{exp}^3)}{N\frac{\pi}{6}d_i^3(1-x)}\right)^{\frac{1}{3}} = d_{exp} \times \left(\frac{1}{(1-x)}\right)^{\frac{1}{3}}$$

For synthesis 2, we have d_i = 6.1 nm, d_{exp} = 8.4 nm, d_f = 11.5 nm, we can calculate the dissolved fraction, x =61%

Therefore 61% of the initial thin shell HgSe/CdS QDs dissolve and deposit as a HgCdSSe shell on the remaining QDs.

Doing a similar calculation for the second aliquot of QDs, we have have d_i = 6.1 nm, d_{exp} = 10.5nm, d_f = 15.3 nm, we get x =68%. This shows that a majority of the dissolution of the QDs occurs during the initial stages of the thick shell synthesis.

The UV-Vis absorption spectra of HgSe/CdS QDs (Fig. S3A(C)) from synthesis 2 show a broad red tail in the shell absorption beyond the CdS band edge, consistent with presence of an alloyed HgCdSSe shell. In contrast, the absorption spectra of samples from synthesis 1 show a sharp shell absorption edge, similar to the absorption of CdS QDs. The shell absorbances in synthesis 2 also have a larger intensity than synthesis 1, consistent with the larger sizes measured by TEM.

Despite the significant dissolution of QDs in synthesis 2, the FTIR spectra of HgSe/CdS QDs from synthesis 2 is similar to those from synthesis 1 (Fig. S3A(D)). This is expected, since 220°C is much higher than the thermal stability window of HgSe, and the ripening dots dissolve nearly instantaneously. Hence none of the cores of the dissolving QDs survive, and the infrared properties of the HgSe/CdS QDs remain unaffected by the dissolution occurring during the synthesis.

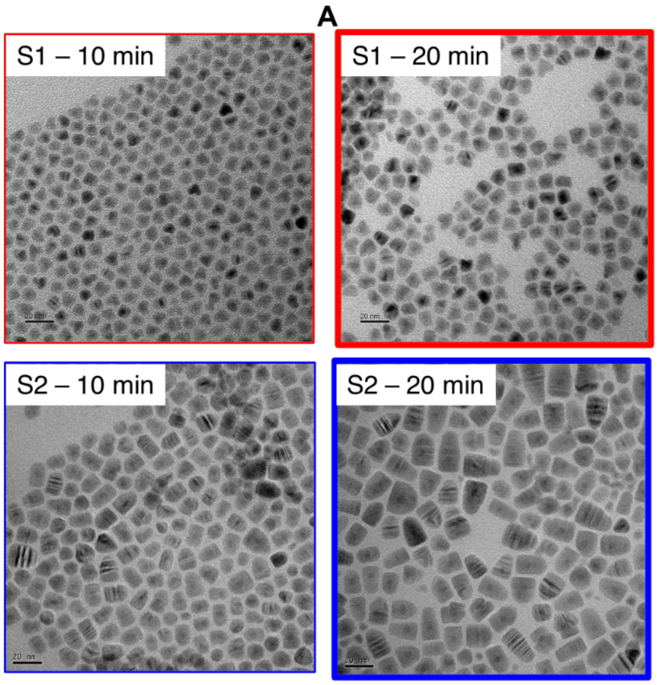

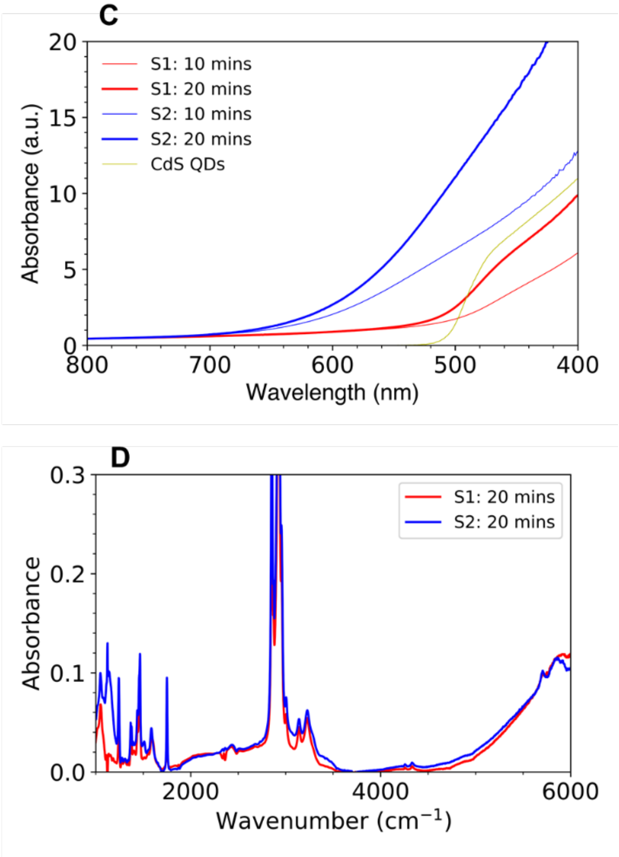

B

Sample	Expected diameter	Measured diameter
S1 – 10 mins	8.2 nm	8.9 nm
**S1 – 20mins**	**10.2 nm**	**10.5 nm**
S2 – 10mins	8.4 nm	11.5 nm
**S2 – 20mins**	**10.5 nm**	**15.3 nm**

Fig. S3A: (A) TEM images of thick shell HgSe/CdS QDs for syntheses (red) 1 and (blue) 2. Table (B) shows a negligible QD dissolution in synthesis 1 and the measured core/shell diameters match well with the expected diameters. Synthesis 2 shows a significant QD dissolution and the core/shell sizes are much larger than the expected size. (C) UV-Visible absorption spectra of HgSe/CdS QDs from (red) synthesis 1 and (blue) synthesis 2, normalized to absorbance at 808 nm. The absorbance of samples from synthesis 2 are much larger than synthesis 1. The broad absorption for samples from syntheses 2 confirm the presence of an alloyed HgCdSSe shell. Absorption spectrum of CdS QDs (yellow) is shown for reference. (D) FTIR spectra of HgSe/CdS QDs from syntheses 1 and 2 after 20 mins of reaction, showing nearly identical absorption features from the HgSe cores.

3B: Radiative and nonradiative lifetimes of HgSe and HgSe/CdS QDs

The radiative lifetime τ_R of a nanoparticle dipole transition is[12–15]

$$\frac{1}{\tau_R} = \frac{\omega^3 p^2}{3\pi\epsilon_0 \hbar c^3} F$$

Here ω is the angular frequency, c is the vacuum light speed, $\hbar$ is the reduced Planck's constant, p is the transition dipole matrix element and ϵ_0 is the vacuum permittivity. The quantity F is a dielectric factor which accounts for electric field screening and the photon density of states in the medium, and $F = \sqrt{\epsilon_2}\left(\frac{3\epsilon_2}{\epsilon_1+2\epsilon_2}\right)^2$ for a nanoparticle of real optical dielectric constant ϵ_1 in a host of real optical dielectric constant ϵ_2. For the intraband transition in HgSe, we calculate the dipole matrix element as

$$p = e\langle 1S_e|r|1P_e\rangle\langle u_{c,S}|u_{c,P}\rangle$$

where e is the electron charge, r is the position operator, $1S_e$ and $1P_e$ are the radial envelope functions, and $u_{c,S}$ and $u_{c,P}$ are the conduction Bloch functions at wavevectors $k_S = \pi/R$ and $k_P = 4.49/R$. A two-band Kane model gives $\langle u_{c,S}|u_{c,P}\rangle \approx 1$ and spherical Bessel envelope functions give $\langle 1S_e|r|1P_e\rangle = 0.306R$.[16] The Kane result may somewhat overestimate the Bloch overlap based on a comparison to tight-binding calculations on the similar HgTe system,[17] and the calculated lifetime is therefore an upper limit. Using the material parameters in Table S3B and emission frequency as 2050 cm^{-1} for comparison with lifetime measurements, this gives a radiative lifetime of 900 ns. Taking into account the standard deviation uncertainty in measurement frequency (2050 ± 20) cm^{-1} and ϵ_1 (10 ± 2), we get the core radiative lifetime (900 ± 300) ns. From this calculated radiative lifetime and the measured intraband PLQY of 7.5×10^{-4} for HgSe QDs, the nonradiative lifetime is 700 ps, with a one-standard deviation range of 0.40 – 1.1 ns.

Quantity	Value
ϵ_1: optical dielectric constant of HgSe QDs	10 ± 2[18,19]
ϵ_2: optical dielectric constant of TCE	2.25
ϵ_S: optical dielectric constant of CdS shell	5.3[20]
R: Radius of HgSe core	2.4 nm
ω for emission at 2050 cm^{-1}	3.9×10^{14} rad/s

Table S3B: Material parameters used for calculation of radiative rate in HgSe and HgSe/CdS QDs.

Growth of the CdS shell leads to a change in the dielectric screening and a decrease in the radiative lifetime. To calculate F for this system, we performed an electrostatic analysis of a core/shell structure modeled as nested dielectric spheres in a dielectric matrix.[21,22] For a core/shell nanocrystal with core radius of R_1 and total radius of R_2, the radiative rate is found to be:

$$\frac{1}{\tau_R} = \frac{\omega^3 p^2}{3\pi\epsilon_0 \hbar c^3} \times \sqrt{\epsilon_2}\left(\left(\frac{R_2}{R_1}\right)^3 \left(\frac{9\,\epsilon_s\,\epsilon_2}{\left(\frac{R_2}{R_1}\right)^3 [(\epsilon_s + 2\epsilon_2)(\epsilon_1 + 2\epsilon_s)] - 2(\epsilon_2 - \epsilon_s)(\epsilon_1 - \epsilon_s)}\right)\right)^2$$

where ϵ_s is the shell optical dielectric constant. The radiative lifetime τ_R as a function of core/shell diameter is plotted in Fig. S3B. The radiative lifetime approaches a value of 700 ns at diameters greater than 8 nm. Taking into account the uncertainties in frequency and ϵ_1, we get the core/shell lifetime to be (700 ± 160). We can therefore use this radiative lifetime for all thick shell HgSe/CdS QDs. From this calculated lifetime and the measured intraband PLQY of 2.1×10^{-2} for thick shell HgSe/CdS QDs, the nonradiative lifetime is 15 ns, with a one-standard deviation range of 7.7 – 28 ns.

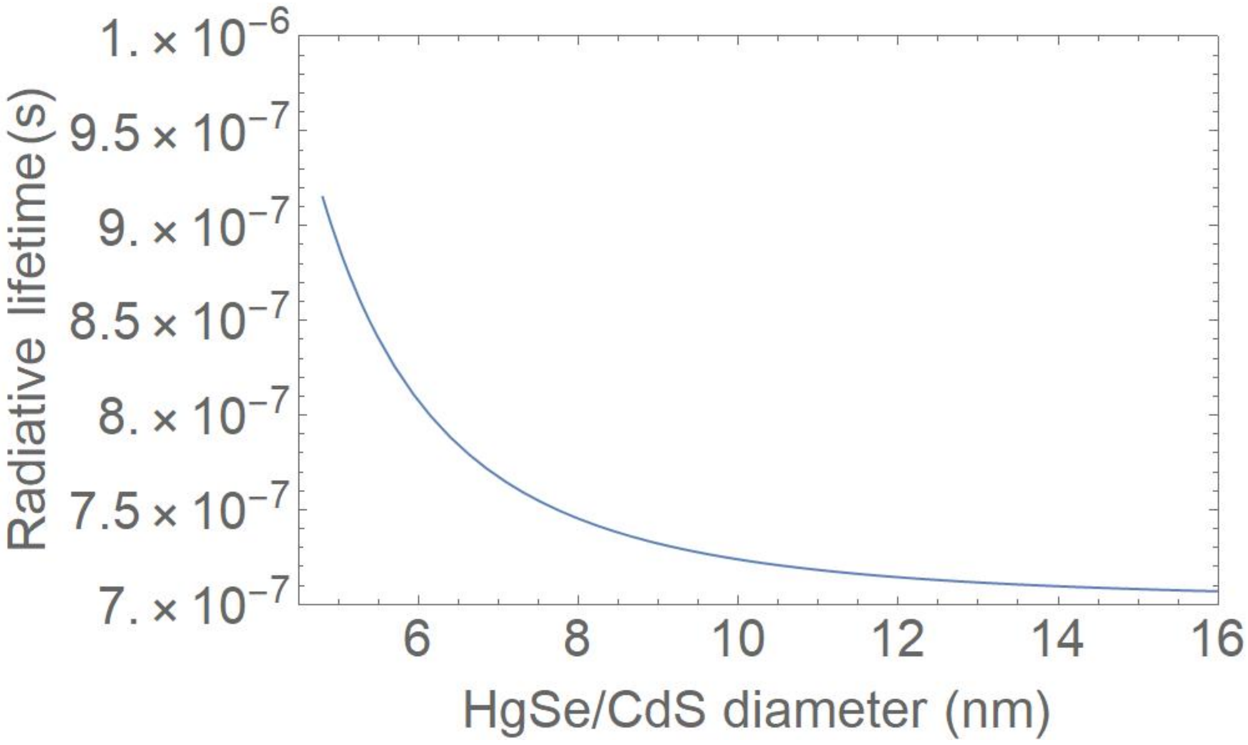

Fig. S3B: Radiative lifetime of HgSe/CdS QDs with a 4.8 nm diameter core, as a function of core/shell diameter. At diameters larger than 8nm, the radiative lifetime saturates to a value around 700 ns.

3C: Measurement of average diameter of bullet-shaped HgSe/CdS QDs

HgSe/CdS QDs develop an anisotropic bullet-shape for HgSe/CdS QDs at large shell thicknesses. To estimate the 'average' diameter, we assumed the nanocrystals to have the same volume as an ellipsoid with two diameters as the short length of the bullet (Fig. S3C(B)) and the third diameter as the long axis of the bullet (Fig. S3C(A)). The volume of the nanobullet was then approximated as $\frac{\pi}{6}d_1^2 d_2$, where d_1 is the bullet diameter and d_2 is the axis length. Nanocrystals appearing as circles in the TEM (Fig. S3C(C)) were assumed to be bullets lying on the short face perpendicular to the axis. The diameter of the circles were thus taken as the bullet diameter d_2. The average d_1 and d_2 values were measured taking an average of 36 values each, and the average diameter was calculated as $d_{average} = (d_1^2 d_2)^{\frac{1}{3}}$.

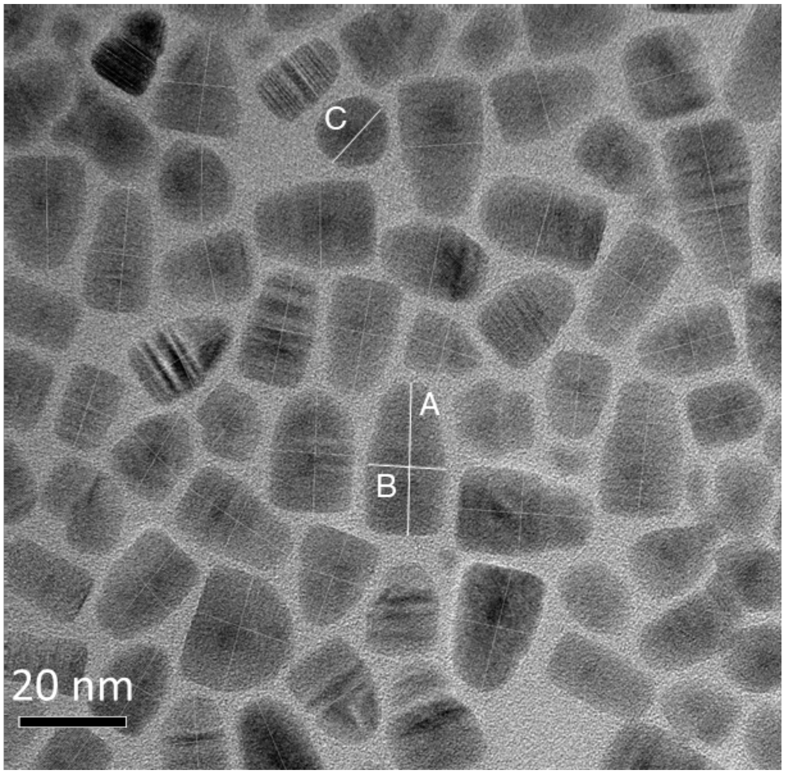

Fig. S3C: Determining the average diameter of bullet-shaped HgSe/CdS QDs.

3D: Calculating the distribution of $1S_e(0)$, $1S_e(1)$ and $1S_e(2)$ QDs

The average $1S_e$ occupancy (N_e) of HgSe in the core or core/shell QDs depends on the position of the Fermi level E_f, determined by the Fermi-Dirac distribution:

$$N_e = 2 \times \left(\frac{1}{e^{\frac{(E_{1S_e} - E_f)}{k_B T}} + 1} \right)$$

Assuming negligible electronic correlation, electrons can occupy the two spin states of $1S_e$ independently. The fraction of QDs with 0, 1 and 2 electrons in $1S_e$ then follows a binomial distribution:

$$1S_e(0)\ fraction = \left(1 - \left(\frac{\mathrm{N}_e}{2}\right)\right)^2$$

$$1S_e(1)\ fraction = 2\left(\frac{N_e}{2}\right) \times \left(1 - \left(\frac{N_e}{2}\right)\right)$$

$$1S_e(2)\ fraction = \left(\frac{N_e}{2}\right)^2$$

A visual schematic of the distribution of QDs is shown in Fig. S3D for different Fermi level positions.

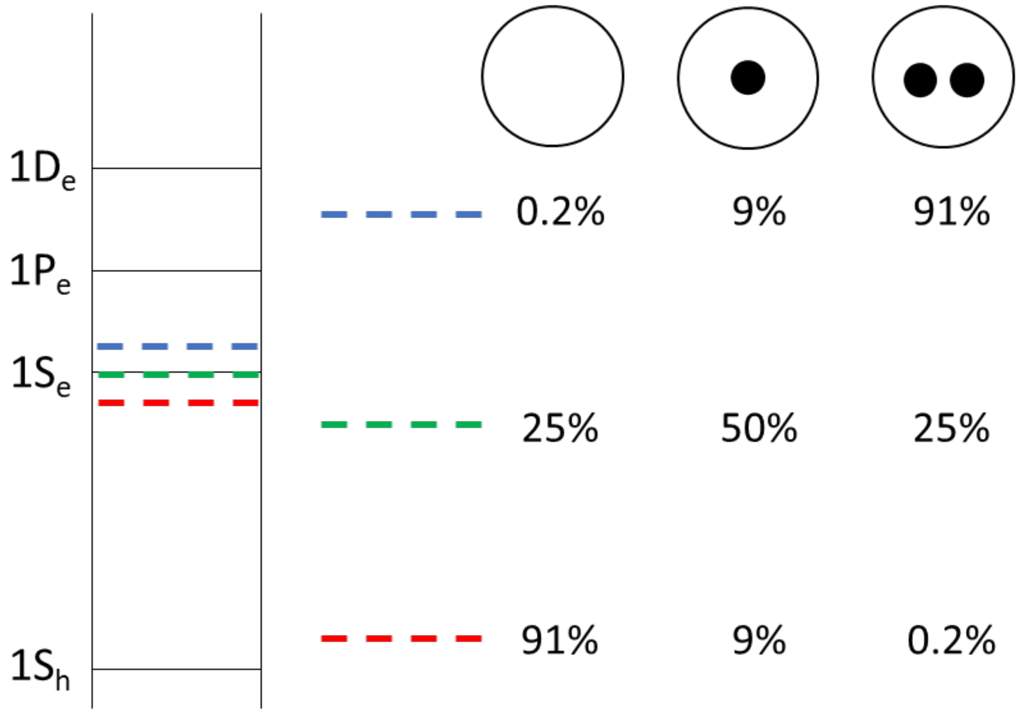

Fig. S3D: Distribution of QDs in $1S_e(0)$, $1S_e(1)$ and $1S_e(2)$ configurations in HgSe at different Fermi level positions (dashed lines).

3E: Fitting of intraband and interband absorptions

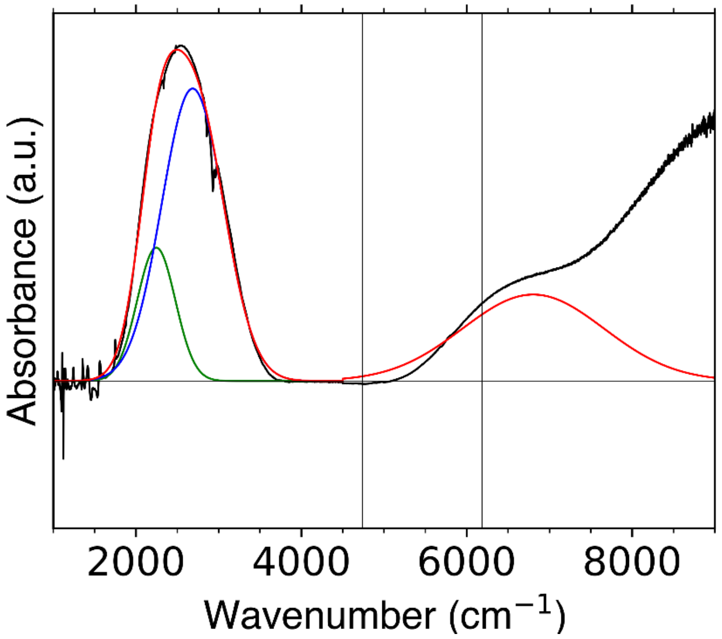

Fig. S3E: Fitting of intraband and interband spectra in thick shell HgSe/CdS QDs. The FTIR measurement is the black curve, while the red curves are fits. The intraband absorption was fit to a sum of gaussians (blue, green) due to the conduction band spin-orbit splitting,[6] while the interband was fit to a single Gaussian. Ratio of areas of intraband and interband absorptions is 1.3 : 1, confirming the similar oscillator strengths for the two transitions. The fitting range for the interband absorption is shown by the vertical lines.

3F: Fermi level-dependent defect-assisted nonradiative relaxation in thick shell HgSe/CdS QDs

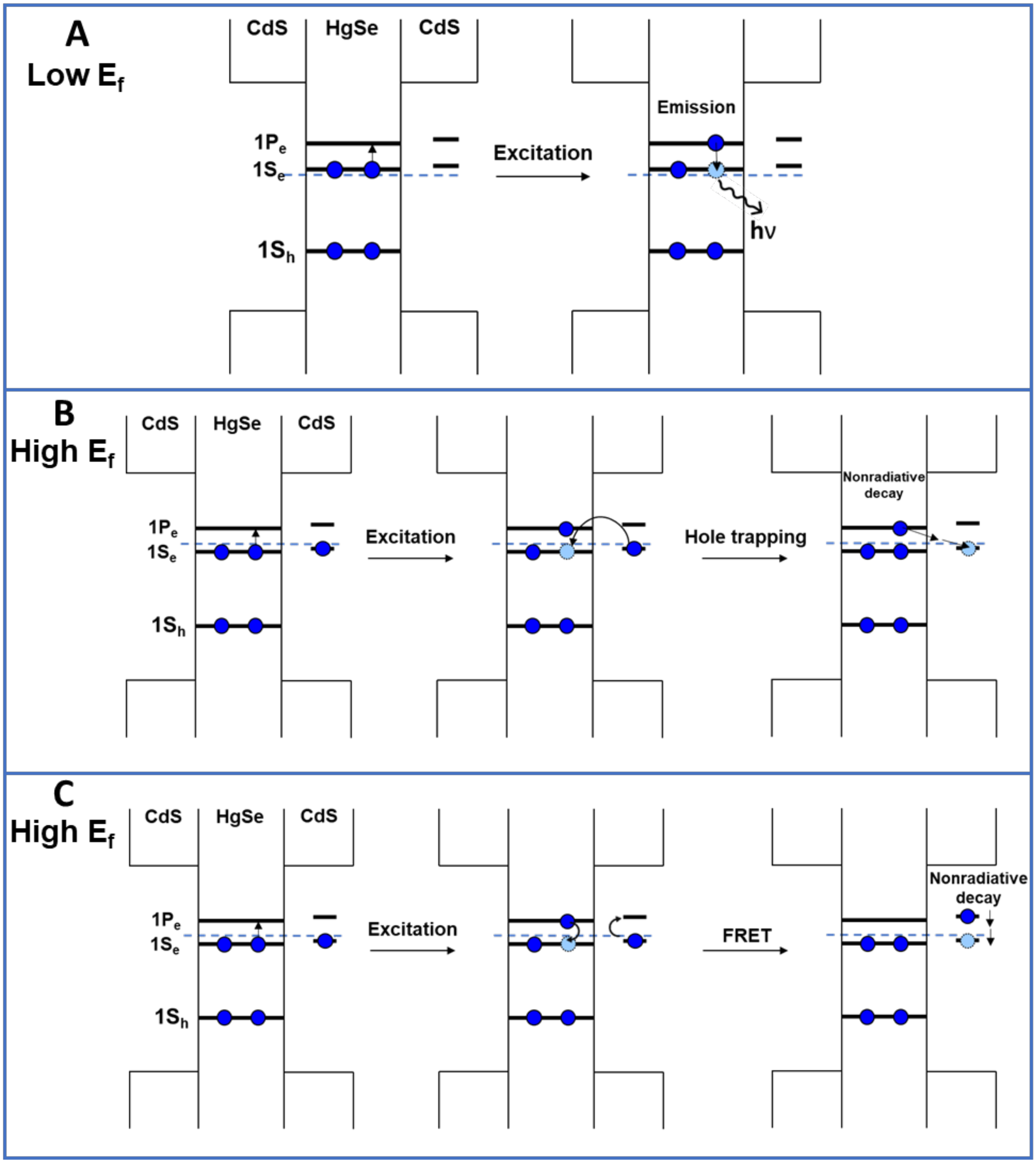

Fig. S3F: Fermi level-dependent defect-assisted nonradiative relaxation in HgSe/CdS QDs. Fermi level is denoted by the dashed blue line. Proposed mechanism of Fermi level-dependent PLQY for thick shell HgSe/CdS QDs at **(A)** low Fermi levels and **(B, C)** high Fermi levels. The CdS shell might

have localized defect states (two levels are depicted) that are close to the $1S_e$ and $1P_e$ states of HgSe. **(A)** When the Fermi level is low, the defect states are empty, and the QDs display intraband photoluminescence on excitation. **(B, C)** When the Fermi level is high, the defect states get filled. When the QD is photoexcited, the filled defect state could introduce a nonradiative decay channel either by **(B)** hole trapping or by **(C)** near-field energy transfer (FRET). Bulk CdS is known to absorb infrared light by transitions between defect states.[23,24] This absorption might be too weak to measure by FTIR, but the close proximity of the core to the shell can lead to a significant nonradiative decay.

4. References

(1) Humeres, E.; Debacher, N. A.; Franco, J. D.; Lee, B. S.; Martendal, A. Mechanisms of Acid Decomposition of Dithiocarbamates. 3. Aryldithiocarbamates and the Torsional Effect. *J. Org. Chem.* **2002**, *67*, 3662–3667.

(2) Morrison, C. E.; Wang, F.; Rath, N. P.; Wieliczka, B. M.; Loomis, R. A.; Buhro, W. E. Cadmium Bis(Phenyldithiocarbamate) as a Nanocrystal Shell-Growth Precursor. *Inorg. Chem.* **2017**, *56*, 12920–12929.

(3) Kim, T.; Jung, Y. K.; Lee, J.-K. The Formation Mechanism of CdSe QDs through the Thermolysis of Cd(Oleate)2 and TOPSe in the Presence of Alkylamine. *J. Mater. Chem. C* **2014**, *2*, 5593–5600.

(4) Analogies, C. S.-; Frameworks, A.; Dance, I. G.; Garbutt, R. G.; Craig, D. C.; Scudder, M. L. The Different Nonmolecular Polyadamantanoid Crystal Structures of Cd(SPh)2 and Cd(SC6H4Me-4)2: Analogies with Microporous Aluminosilicate Frameworks1. *Inorg. Chem.* **1987**, *26*, 4057–4064.

(5) Nan, W.; Niu, Y.; Qin, H.; Cui, F.; Yang, Y.; Lai, R.; Lin, W.; Peng, X. Crystal Structure Control of Zinc-Blende CdSe/CdS Core/Shell Nanocrystals: Synthesis and Structure-Dependent Optical Properties. *J. Am. Chem. Soc.* **2012**, *134*, 19685–19693.

(6) Melnychuk, C.; Guyot-Sionnest, P. Auger Suppression in N-Type HgSe Colloidal Quantum Dots. *ACS Nano* **2019**, *13*, 10512–10519.

(7) Shen, G.; Guyot-sionnest, P. HgTe/CdTe and HgSe/CdX (X = S, Se, and Te) Core/Shell Mid-Infrared Quantum Dots. *Chem. Mater.* **2019**, *31*, 286–293.

(8) Niu, Y.; Pu, C.; Lai, R.; Meng, R.; Lin, W.; Qin, H.; Peng, X. One-Pot/Three-Step Synthesis of Zinc-Blende CdSe/CdS Core/Shell Nanocrystals with Thick Shells. *Nano Res.* **2017**, *10*, 1149–1162.

(9) Qin, H.; Niu, Y.; Meng, R.; Lin, X.; Lai, R.; Fang, W.; Peng, X. Single-Dot Spectroscopy of Zinc-Blende CdSe/CdS Core/Shell Nanocrystals: Nonblinking and Correlation with Ensemble Measurements. *J. Am. Chem. Soc.* **2014**, *136*, 179–187.

(10) Zhou, J.; Zhu, M.; Meng, R.; Qin, H.; Peng, X. Ideal CdSe/CdS Core/Shell Nanocrystals Enabled by Entropic Ligands and Their Core Size-, Shell Thickness-, and Ligand-Dependent Photoluminescence Properties. *J. Am. Chem. Soc.* **2017**, *139*, 16556–16567.

(11) Jeong, K. S.; Deng, Z.; Keuleyan, S.; Liu, H.; Guyot-Sionnest, P. Air-Stable n-Doped Colloidal HgS Quantum Dots. *J. Phys. Chem. Lett.* **2014**, *5*, 1139–1143.

(12) de Vries, P.; Lagendijk, A. Resonant Scattering and Spontaneous Emission in Dielectrics: Microscopic Derivation of Local-Field Effects. *Phys. Rev. Lett.* **1998**, *81*, 1381–1384.

(13) Yablonovitch, E.; Gmitter, T. J.; Bhat, R. Inhibited and Enhanced Spontaneous Emission from Optically Thin AlGaAs/GaAs Double Heterostructures. *Phys. Rev. Lett.* **1988**, *61*, 2546–2549.

(14) Hilborn, R. C. Einstein Coefficients, Cross Sections, f Values, Dipole Moments, and All That. *Am. J. Phys.* **1982**, *50*, 982–986.

(15) Toptygin, D. Effects of the Solvent Refractive Index and Its Dispersion on the Radiative Decay Rate and Extinction Coefficient of a Fluorescent Solute. *J. Fluoresc.* **2003**, *13*, 201–219.

(16) Guyot-Sionnest, P.; Hines, M. A. Intraband Transitions in Semiconductor Nanocrystals. *Appl. Phys. Lett.* **1998**, *72*, 686–688.

(17) Allan, G.; Delerue, C. Tight-Binding Calculations of the Optical Properties of HgTe Nanocrystals. *Phys. Rev. B* **2012**, *86*, 165437.

(18) Einfeldt, S.; Goschenhofer, F.; Becker, C. R.; Landwehr, G. Optical Properties of HgSe. *Phys. Rev. B* **1995**.

(19) Szuszkiewicz, W.; Witowski, A. M.; Grynberg, M. The Dynamic Dielectric Function in HgSe and

HgTe. *Phys. Status Solidi* **1978**, *87* (2), 637–645.

(20) Ninomiya, S.; Adachi, S. Optical Properties of Wurtzite CdS. *J. Appl. Phys.* **1995**, *78* (2), 1183–1190.

(21) Duan, C. K.; Reid, M. F. Macroscopic Models for the Radiative Relaxation Lifetime of Luminescent Centers Embedded in Surrounding Media. *Spectrosc. Lett.* **2007**, *40* (2), 237–246.

(22) Zangwill, A. *Modern Electrodynamics*; Cambridge University Press, 2012.

(23) Tutihasi, S. Quenching of Photoconductivity in Cadmium Sulfide. *J. Opt. Soc. Am.* **1956**, *46*, 443.

(24) Kulp, B. A. Defects in Cadmium Sulfide Crystals. *J. Appl. Phys.* **1965**, *36*, 553–558.
